# Hypoxia-induced ZEB1 promotes cervical cancer immune evasion by strengthening the CD47-SIRPα axis

**DOI:** 10.1186/s12964-023-01450-4

**Published:** 2024-01-05

**Authors:** Xiao-Jing Chen, Chu-Hong Guo, Zi-Ci Wang, Yang Yang, Yu-Hua Pan, Jie-Ying Liang, Mei-Ge Sun, Liang-Sheng Fan, Li Liang, Wei Wang

**Affiliations:** 1 Guangzhou Medical University/Department of Obstetrics and Gynecology, The First Affiliated Hospital of Guangzhou Medical University, 151 Yanjiang West Road, Yuexiu District, Guangzhou, 510120 People’s Republic of China; 2 Department of Pathology, Nanfang Hospital, Southern Medical University, 1838 Guangzhou Avenue North, Baiyun District, Guangzhou, 510515 People’s Republic of China

**Keywords:** Cervical cancer, Hypoxia, Immune evasion, Exosome, CD47-SIRPα axis

## Abstract

**Background:**

The dynamic interaction between cancer cells and tumour-associated macrophages (TAMs) in the hypoxic tumour microenvironment (TME) is an active barrier to the effector arm of the antitumour immune response. Cancer-secreted exosomes are emerging mediators of this cancer-stromal cross-talk in the TME; however, the mechanisms underlying this interaction remain unclear.

**Methods:**

Exosomes were isolated with ExoQuick exosome precipitation solution. The polarizing effect of TAMs was evaluated by flow cytometry, western blot analysis, immunofluorescence staining and in vitro phagocytosis assays. Clinical cervical cancer specimens and an in vivo xenograft model were also employed.

**Results:**

Our previous study showed that hypoxia increased the expression of ZEB1 in cervical squamous cell carcinoma (CSCC) cells, which resulted in increased infiltration of TAMs. Here, we found that hypoxia-induced ZEB1 expression is closely correlated with CD47-SIRPα axis activity in CSCC, which enables cancer cells to evade phagocytosis by macrophages and promotes tumour progression. ZEB1 was found to directly activate the transcription of the CD47 gene in hypoxic CSCC cells. We further showed that endogenous ZEB1 was characteristically enriched in hypoxic CSCC cell-derived exosomes and transferred into macrophages via these exosomes to promote SIRPα^+^ TAM polarization. Intriguingly, exosomal ZEB1 retained transcriptional activity and reprogrammed SIRPα^+^ TAMs via activation of the STAT3 signalling pathway in vitro and in vivo. STAT3 inhibition reduced the polarizing effect induced by exosomal ZEB1. Knockdown of ZEB1 increased the phagocytosis of CSCC cells by macrophages via decreasing CD47 and SIRPα expression.

**Conclusions:**

Our results suggest that hypoxia-induced ZEB1 promotes immune evasion in CSCC by strengthening the CD47-SIRPα axis. ZEB1-targeted therapy in combination with CD47-SIRPα checkpoint immunotherapy may improve the outcomes of CSCC patients in part by disinhibiting innate immunity.

**Supplementary Information:**

The online version contains supplementary material available at 10.1186/s12964-023-01450-4.

## Background

Cancer immunotherapy is becoming established as a powerful tool for combating cancer and is achieving success in clinical trials for multiple cancer types [1]. However, for the majority of patients with cervical squamous cell carcinoma (CSCC), little or no progress has been made, presumably due to the characteristics of hypoxia that shape the local immunosuppressive tumour microenvironment (TME) and thus limit the effectiveness of antitumour immune responses [2]. Therefore, new strategies that can efficiently reprogram the immunosuppressive hypoxic TME and further elicit antitumour immunity are urgently needed.

Hypoxia induces immune evasion through various mechanisms [3]. A major mechanism by which cancer cells evade the innate immune system is the expression of CD47, which is a cell surface protein that interacts with signal regulatory protein α (SIRPα) on the surface of macrophages to block phagocytosis [4, 5]. Despite the considerable enthusiasm for targeting CD47 for cancer immunotherapy [6, 7], remarkably, we know very little about transcriptional regulation of the CD47 gene. Intratumoural hypoxia is a common finding in solid tumours [8]. The hypoxia-inducing factors HIF-1α and HIF-2α can regulate the expression of immune checkpoints such as PD-L1 [9]. Analysis of a human breast cancer database revealed that high CD47 expression is also correlated with increased HIF target gene expression and decreased patient survival [10]. Here, we report that CD47 expression is induced in a ZEB1-dependent manner when CSCC cells are exposed to hypoxia. To overcome this immunological sanctuary, further mechanisms of the CD47-SIRPα checkpoint in the hypoxic TME need to be investigated.

Hypoxic stress in solid tumours stimulates the secretion of nanosized membrane-encapsulated vesicles (30–150 nm in diameter) known as exosomes that promote angiogenesis, metastasis, and host immunosuppression to drive tumour progression [11, 12]. Exosomes play an essential role in intercellular communication by transporting functional biomolecules, such as proteins, lipids, RNAs, and DNAs, and the exosomal cargo differs depending on the source cell [13]. It has recently been found that exosomes are endogenous carriers of protumorigenic factors that participate in oncogenesis [14]. However, whether oncogenic transcription factors are transferred by exosomes is unknown. Our previous study identified high expression of ZEB1 in hypoxic CSCC cells [15]. ZEB1 is a key element of a network of transcription factors that endows cancer cells with a pro-invasive and mesenchymal-like phenotype and predicts poor clinical outcomes in a variety of human cancers [16]. Over the past decade, extensive studies have highlighted unsuspected intrinsic oncogenic functions of ZEB1 that impact tumorigenesis from its earliest stages [17]. In this study, we found that endogenous ZEB1 was characteristically enriched in hypoxic CSCC cell-derived exosomes and transferred into macrophages via these exosomes. Intriguingly, ZEB1 expression was also closely correlated with CD47-SIRPα axis activity in the hypoxic TME. Although our previous study reported that ZEB1 facilitates tumour-associated macrophage (TAM) infiltration, its roles in hypoxia-induced immune evasion need to be further investigated.

Here, we demonstrate that ZEB1 activates the transcription of the CD47 gene in hypoxic CSCC cells. Moreover, exosomal intercellular transfer of transcriptionally active ZEB1 was correlated with SIRPα^+^ TAM polarization. We propose that hypoxia-induced ZEB1 promotes immune evasion in CSCC by increasing the activity of the CD47-SIRPα axis. The unexpected role of ZEB1 established by our study offers additional approaches for future cancer immunotherapy.

## Materials and methods

### Cell lines and culture

Human CSCC cell lines (SiHa and C33a) and the human monocytic cell line THP-1 were purchased from the American Type Culture Collection (ATCC, USA) and cultured according to the provider’s guidelines. Hypoxia exposure was performed by placing cells in a hypoxic incubator (Thermo Fisher Scientific, USA) maintained at low oxygen tension (1% O_2_, 5% CO_2_, and 94% N_2_). Normoxia exposure was performed by placing cells in a normoxic incubator (21% O_2_, 5% CO_2_, and 74% N_2_).

### Clinical specimens

A total of 20 archived formalin-fixed, paraffin-embedded CSCC specimens were obtained from the Department of Gynecological Oncology of The First Affiliated Hospital of Guangzhou Medical University between 2012 and 2014 (Supplemental Table 3). All samples were primary tumour samples obtained from CSCC patients who underwent abdominal radical hysterectomy without prior radiotherapy or chemotherapy. Carbonic anhydrase IX (CAIX) is an established cellular biomarker of hypoxia [18, 19] and was used to evaluate the hypoxic state of tumour tissues in patients. The study was approved by the Institutional Research Ethics Committee of The First Affiliated Hospital of Guangzhou Medical University (No. 2022-K-15). As the specimens included in the study were derived from residual specimens and the corresponding patient information was obtained from routine medical records, our study met the requirement of exemption from informed consent. Each section was evaluated by two experienced pathologists who were blinded to the clinicopathological data of the patients.

### Exosome isolation and characterization

Exosomes were isolated from cells and characterized as previously described [11, 12]. Briefly, exosomes were separated by mixing 10 mL of cellular conditioned medium (CM) with ExoQuick Exosome Precipitation Solution (EZBioscience, USA) according to the manufacturer's protocol. After incubation at 4 °C overnight, the mixture was centrifuged at 10,000 × g for 30 min. Pelleted exosomes were resuspended in 100 µl of PBS and subjected to several experiments, including morphological identification by transmission electron microscopy (TEM), size identification by nanoparticle tracking analysis (NTA), protein analysis, in vitro treatment, and in vivo administration. For TEM, exosomes were fixed with 2% glutaraldehyde, stained with negative-contrast phosphotungstic acid, and imaged with a transmission electron microscope (Hitachi, Japan). For protein analysis, exosome preparations were evaluated using the BCA Protein Assay Kit (Beyotime, China). For in vitro treatment, 1 × 10^5^ recipient cells were treated with 10 µg of exosomes resuspended in 100 µl of phosphate-buffered saline (PBS) for 48 h [11]. For in vivo administration, 10 µg of exosomes resuspended in 20 µl of PBS was injected into the centre of tumours every other day [12].

### THP-1 cell differentiation

THP-1 cell differentiation was induced by treatment with 100 ng/ml phorbol 12-myristate 13-acetate (PMA, Sigma‒Aldrich) in DMSO for 6 h. Cells were then polarized towards the M2 phenotype by incubation for 48 h with CSCC cell-derived exosomes (10 µg) in the presence of PMA. Cells used for the control group were maintained in the presence of PMA for 48 h in normal growth medium without exosomes.

### Exosome uptake assay

For the exosome uptake assay, PKH67 membrane dye (Sigma, USA) was added to PBS at a 1 µM concentration and used to label exosomes for 20 min before washing. Excess dye was removed by an additional washing step, and labelled exosomes (10 µg) were resuspended and used to treat THP-1 cells. After a 48-h incubation, THP-1 cells were then labelled with phalloidin and imaged by confocal microscopy.

### Establishment of cell lines with stable ZEB1 overexpression and ZEB1 silencing

Lentiviruses containing the ZEB1 overexpression plasmid (ZEB1) and the corresponding negative control (NC) plasmid or the ZEB1 knockdown shRNA (sh-ZEB1) and the corresponding negative control shRNA (sh-NC) were all purchased from GeneChem, Inc. (Shanghai, P. R. China). SiHa and C33a cells were transfected with ZEB1 overexpression or knockdown lentivirus at a multiplicity of infection (MOI) of 10, and selection was then performed by incubation in the presence of 2.5 µg/ml puromycin for 2 weeks. The sequences used for ZEB1 overexpression, ZEB1 silencing, and the respective NC sequences are provided in Supplemental Table 1.

### RNA extraction and RT‒qPCR

RNA was extracted from cell lines with TRIzol Reagent (Solarbio, China). RT‒qPCR was performed as previously described [20]. The primers specific for ZEB1, CD47, and β-actin were purchased from Invitrogen. The sequences of the primers used were as follows: ZEB1 primers, forward: 5’-CAGCTTGATACCTGTGAATGGG-3’ and reverse: 5’-TATCTGTGGTCGTGTGG GACT-3’; CD47 primers, forward: 5’-TCCGGTGGTATGGATGAGAAA-3’ and reverse: 5’-ACCAAGGCCAGTAGCATTCTT-3’; and β-actin primers, forward: 5’-CTGGGCTACACTGAG CACC-3’ and reverse: 5’-AAGTGGTCGTTGAGGGCA ATG-3’. Target mRNA expression was normalized to β-actin mRNA expression.

### Flow cytometry (FCM)

Cells were stained with an APC-conjugated anti-CD47 (#CC2C6, BioLegend, USA) antibody according to the manufacturer's instructions. Briefly, staining was performed at 4 °C for 30 min in PBS in the dark. After washing, samples were immediately analysed by FCM using an Attune® Acoustic Focusing Cytometer (Life Technologies, USA). The acquired data were analysed using FlowJo software (TreeStar, USA).

### Western blot analysis

Western blot analysis was performed as previously described [15]. The primary antibodies used were as follows: anti-ZEB1 (#70,512, CST, USA), anti-CD47 (#63,000, CST, USA), anti-SIRPα (#13,379, CST, USA), anti-CD163 (#ab156769, Abcam, UK), anti-STAT3 (#D1A5, CST, USA), anti-phosphorylated (p)-STAT3 (#D3A7, CST, USA), anti-NF-κB (#8242, CST, USA), anti-p-NF-κB (#3033, CST, USA), anti-AKT (#4685, CST, USA), anti-p-AKT (#4060, CST, USA), anti-ERK1/2 (#4695, CST, USA), anti-p-ERK1/2 (#4370, CST, USA), an Exosomal Marker Antibody Sampler Kit (#74,220, CST, USA), anti-Calnexin (#2679, CST, USA) and anti-β-actin (#3700, CST, USA). The secondary antibodies used were horseradish peroxidase-conjugated anti-rabbit (#ab6721, Abcam, UK) and anti-mouse (#ab6789, Abcam, UK) immunoglobulin G.

### Multiplex immunofluorescence staining

Tissue sections were analysed by immunofluorescence staining with the Opal 4-Color Kit (PerkinElmer, USA) as previously described [20]. The primary antibodies used were as follows: anti-CAIX (#ab184006, Abcam, USA), anti-ZEB1 (#70,512, CST, USA), anti-CD47 (#63,000, CST, USA), anti-SIRPα (#13,379, CST, USA), and anti-CD163 (a human TAM marker; #ab156769, Abcam, UK). The sections were mounted in neutral gum and visualized under a fluorescence microscope (Olympus, Japan). Semiquantitative analysis was performed using the H score method [21]. Fields imaged at 400 × magnification were used for H score assessment, and the staining intensity in tumour cells was scored as 0, 1, 2, or 3, corresponding to negative, weak, intermediate, or strong fluorescence staining, respectively. The total number of cells in each field and the number of cells with each staining intensity score were determined. The H score was calculated as follows: (% of cells with a staining intensity score of 1 × 1) + (% of cells with a staining intensity score of 2 × 2) + (% of cells with a staining intensity score of 3 × 3). An H score of between 0 and 300 was obtained, where a score of 300 indicated strong staining in 100% of the tumour cells (3 +). The median H score values were used to separate patients into the low and high expression groups. Patients were also classified into the normoxia group and hypoxia group based on the median H score (expression level = 150) of CAIX, and the locations used to determine the H scores in the multiplex immunofluorescence staining images were selected at random in the tumour area. In addition, based on our previous study and other studies of TAM markers, CD163 is a highly specific macrophage marker that has been suggested to be expressed primarily by protumoral M2-like TAMs [22, 23].

### In vitro phagocytosis assay

THP-1 macrophages were labelled with 5 μM DiI (Life Technologies, USA) in serum-free medium for 2 h and then seeded in a 24-well tissue culture plate at 5 × 10^4^ cells/well. CFSE-labelled CSCC cells (SiHa and C33a) in the different groups were harvested as single-cell suspensions and added to the macrophages at 5 × 10^4^ cells/well. After 4 h of incubation, phagocytic events were imaged with an inverted microscope (LSM 880, Carl Zeiss, Germany). The phagocytosed tumour cells appeared dark under bright field imaging conditions and were colocalized with the red fluorescence of macrophages. The phagocytic index was calculated using the following equation: phagocytic index = number of phagocytosed cells/number of macrophages. At least 200 macrophages were counted per well. All experiments were independently repeated 3 times, and the data were statistically analysed by experienced experimentalists to avoid bias.

### In vivo tumour model

Six-week-old nude mice (20–23 g) were purchased from the Experimental Animal Center, Southern Medical University (Guangzhou, China). The xenograft CSCC model was established in nude mice by inoculation of SiHa cells (5 × 10^6^) into the flank. The tumour size (mm^3^) was measured every 4 days, and the tumour volume was calculated using the following formula: volume = (width)^2^ × length/2. When the tumour volume reached 50 mm^3^, exosomes (10 µg) secreted by SiHa^NC^/Nx, SiHa^ZEB1^/Nx, SiHa^sh−NC^/Hx, or SiHa^sh−ZEB1^/Hx cells were then injected into the tumour centre (*n* = 3/group) every other day for three weeks. After three weeks, the nude mice were euthanized using a barbiturate overdose, and the tumours were collected for analysis. Animal experiments were carried out in accordance with the Guidelines for the Care and Use of Laboratory Animals and approved by the Institutional Review Board of Nanfang Hospital, Southern Medical University (No. NFYY-2016–0230).

### Dual-luciferase reporter assay

The expression of ZEB1 target genes in SiHa and C33a cells was measured by using a dual-luciferase reporter assay according to the manufacturer’s instructions [15]. Briefly, the ZEB1 or control pCDNA3.1( +) vector with the pGL3-CD47 promoter (GeneChem Inc, China) was cotransfected into cells using Lipofectamine™ 2000 (Invitrogen, USA). Luciferase activity was measured 48 h after transfection by the Dual-Luciferase Reporter Assay System. Each assay was repeated 3 times. The cloned sequences are provided in Supplemental Table 2.

### Statistical analysis

SPSS (version 20.0) software was used for statistical analysis. Significance was determined by a *t* test or one-way analysis of variance (ANOVA). Frequency tables were analysed using the chi-squared test, and Pearson correlation analysis was used to assess the significance of correlations between categorical variables. All experiments were performed in triplicate, and quantitative data are presented as the means ± SDs. Differences with *P* < 0.05 were considered to be statistically significant.

## Results

### ZEB1 expression positively correlates with CD47 and SIRPα expression in the hypoxic TME of CSCC

**Fig. 1:**
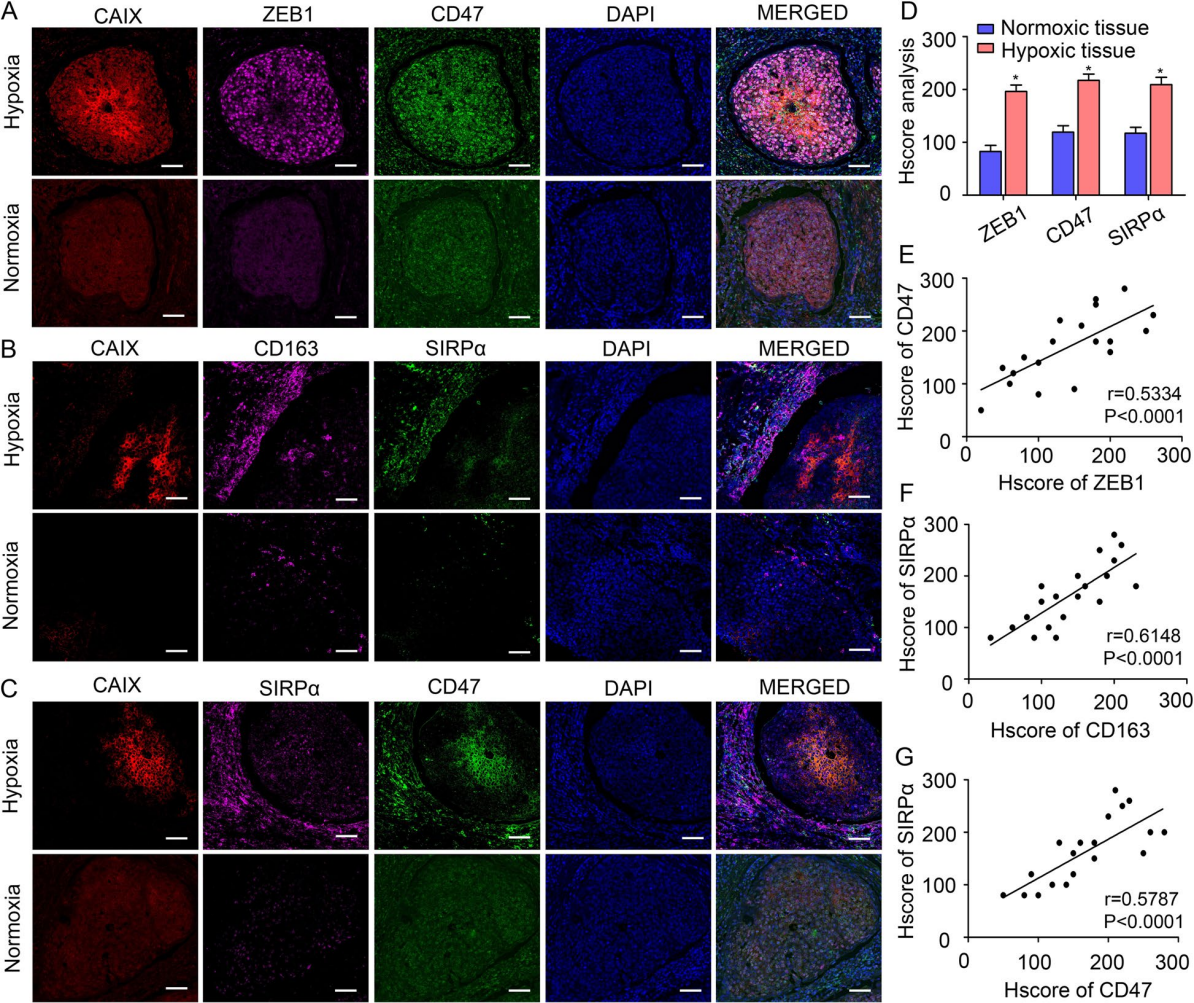
ZEB1 expression positively correlates with CD47 and SIRPα expression in the hypoxic TME of CSCC. **A** Representative images of CAIX (red), ZEB1 (purple), CD47 (green) and DAPI (blue) fluorescence staining in CSCC tissues. **B** Representative images of CAIX (red), CD163^+^ TAMs (purple), SIRPα^+^ TAMs (green) and DAPI (blue) fluorescence staining in CSCC tissues. **C** Representative images of CAIX (red), SIRPα^+^ TAMs (purple), CD47 (green) and DAPI (blue) fluorescence staining in CSCC tissues. Images at 400 × magnification are shown (scale bar, 50 μm). **D** Statistical analysis showing the differences in the expression levels of ZEB1, CD47 and SIRPα between normoxic and hypoxic tissues. *P < 0.05 by Student’s t test. **E**, **G** The clinical relevance of ZEB1, CD47 and SIRPα was evaluated by Pearson correlation analysis

Hypoxia stimulates the expression of multiple genes that enable tumour cells to escape immunosurveillance, leading to patient mortality [4]. Immunofluorescence staining was applied to analyse the expression of carbonic anhydrase IX (CAIX, an established cellular biomarker of hypoxia), ZEB1, CD163 (an M2-like TAM marker in human), CD47 and SIRPα in tissues derived from CSCC patients. As shown in Fig. 1A, intense ZEB1 and CD47 staining was observed in the hypoxic regions where CAIX was abundantly expressed. In contrast, ZEB1 and CD47 were expressed at low levels in CAIX-deficient normoxic regions. ZEB1 and CD47 were colocalized within hypoxic cancer cell islets. In addition, considerable infiltration of CD163^+^ TAMs and SIRPα^+^ TAMs were observed in the stroma surrounding the hypoxic cancer cell islets but not in the normoxic regions. Colocalization of CD163 and SIRPα was also observed, suggesting that SIRPα expression correlates with the M2-like TAM phenotypic switch (Fig. 1B). SIRPα^+^ TAM infiltration was found in the stroma surrounding hypoxic regions with high CD47 expression (Fig. 1C). Statistical analysis showed that the expression levels of ZEB1, CD47 and SIRPα were higher in hypoxic tissues than in normoxic tissues (Fig. 1D, *P* < 0.05). Clinical relevance was evaluated by Pearson correlation analysis, and significant correlations between ZEB1 and CD47 expression, CD163 and SIRPα expression, and CD47 and SIRPα expression were observed (Fig. 1E-G, *r* = 0.5334, *P* < 0.0001; *r* = 0.6148, *P* < 0.0001; *r* = 0.5787, *P* < 0.0001, respectively). Collectively, these results suggest that the expression levels of ZEB1 and CD47 are upregulated in the hypoxic regions of CSCC tissues, which in turn corresponds to greater accumulation of SIRPα^+^ TAMs.

### Hypoxia increases CD47 expression in a ZEB1-dependent manner

**Fig. 2:**
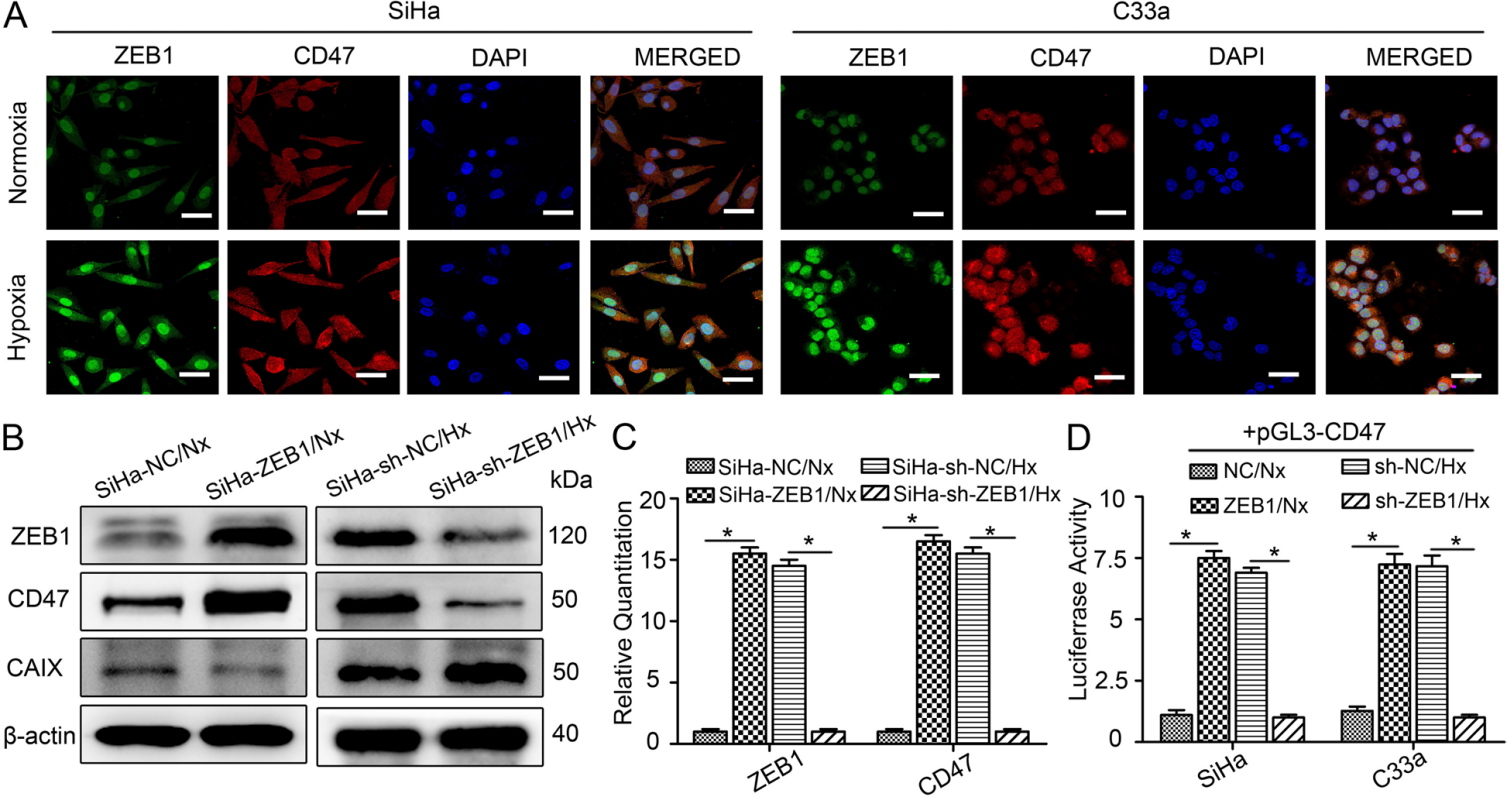
Hypoxia increases CD47 expression in a ZEB1-dependent manner. **A** Representative images of ZEB1 (green), CD47 (red) and DAPI (blue) immunofluorescence staining in normoxic and hypoxic SiHa and C33a cells (scale bar, 50 μm). **B** Western blot analysis results showing the protein levels of ZEB1 and CD47 in SiHa^ZEB1^/Nx, SiHa^NC^/Nx, SiHa^sh−NC^/Hx, and SiHa^sh−ZEB1^/Hx cells. **C** RT‒qPCR analysis results showing the RNA levels of ZEB1 and CD47 in SiHa^ZEB1^/Nx, SiHa^NC^/Nx, SiHa^sh−NC^/Hx, and SiHa^sh−ZEB1^/Hx cells. **D** A dual-luciferase reporter assay system was used to demonstrate the direct binding of ZEB1 to the CD47 promoter region. *P < 0.05. The ZEB1 and NC superscripts indicate the ZEB1 overexpression and control plasmid, respectively. The sh-ZEB1 and sh-NC superscripts indicate the ZEB1 knockdown and negative control shRNAs, respectively. Nx, normoxia; Hx, hypoxia

We analysed the effects of hypoxia on ZEB1 and CD47 expression in CSCC cells (SiHa and C33a) in vitro. SiHa and C33a cells were initially exposed to hypoxic conditions (Hx) for 48 h as previously described [15]. Cells maintained under normoxic conditions (Nx) served as controls. The hypoxic CSCC cells exhibited clear upregulation of ZEB1 and CD47, as visualized by immunofluorescence staining (Fig. 2A). CD47 expression was also detected by flow cytometry, and the results were consistent with the immunofluorescence results (Supplemental Fig. 1). Since ZEB1 is one of the major transcription factors of epithelial-to-mesenchymal transition (EMT), we further investigated whether a morphological switch from an epithelial to a mesenchymal state could be observed in hypoxic CSCC cells. Morphological observation showed that after hypoxia treatment, CSCC cells had significant changes indicative of a mesenchymal morphology, manifested as a long spindle shape and elongated pseudopodia (Supplemental Fig. 2A). In addition, hypoxia increased the expression of N-cadherin, vimentin and snail and decreased the expression of E-cadherin in CSCC cells (Supplemental Fig. 2B). These results suggested that ZEB1 is indeed functional under hypoxia. We then established SiHa and C33a cells with stable overexpression or silencing of ZEB1 to investigate whether ZEB1 is essential for CD47 expression. Western blot analysis revealed that the expression of ZEB1, CD47 and CAIX was highly upregulated at the translational level under hypoxic conditions. Silencing or overexpressing ZEB1 significantly decreased or increased CD47 expression, respectively (Fig. 2B for SiHa cells, Supplemental Fig. 3A for C33a cells). The RT‒qPCR results were consistent with the western blot results (Fig. 2C for SiHa cells, Supplemental Fig. 3B for C33a cells,* P* < 0.05). To uncover the molecular mechanisms of ZEB1-induced CD47 expression, a dual-luciferase reporter assay was performed to demonstrate the role of ZEB1 in CD47 expression. As shown in Fig. 2D, silencing or overexpressing ZEB1 significantly decreased or increased the firefly luciferase activity driven by the pGL3-CD47 promoter in both SiHa and C33a cells (*P* < 0.05). Thus, there is a ZEB1-dependent increase in the expression of CD47 when CSCC cells are exposed to hypoxia.

**Fig. 3:**
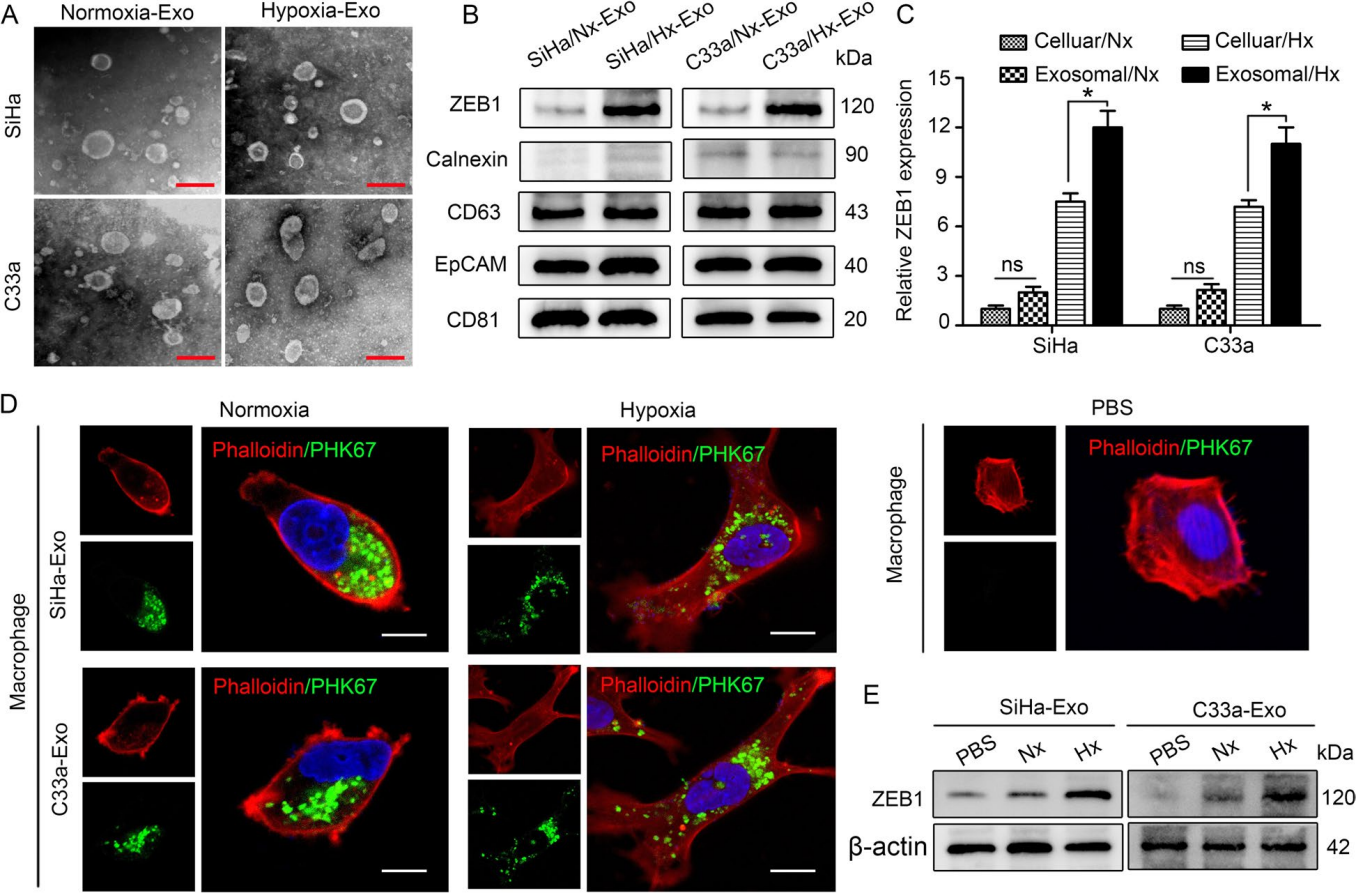
ZEB1 can be enriched in hypoxic CSCC cell-derived exosomes and transferred to macrophages*.* **A** The morphology exosomes secreted from hypoxic/normoxic SiHa and C33a cells was confirmed by transmission electron microscopy (TEM). Scale bar, 100 nm. **B** Positive expression of exosomal markers (CD63, EpCAM and CD81), negative expression of the exosome exclusion marker Calnexin and ZEB1 expression in exosomes was detected by western blotting. **C** ZEB1 expression levels in the indicated cells and paired exosomes were measured by western blotting (relative grayscale analysis). *P < 0.05 by Student’s t test. ns, no significance. **D** THP-1 macrophages pretreated with PKH67-labelled exosomes (green) secreted by hypoxic/normoxic CSCC cells or with PBS for 48 h were stained with phalloidin (red) and DAPI (blue) for confocal microscopy. Scale bar, 20 µm. **E** ZEB1 levels in THP-1 macrophages pretreated with the indicated exosomes or PBS for 48 h were detected by western blotting. *, *P* < 0.05. Exo, exosome; Nx, normoxia; Hx, hypoxia

### ZEB1 can be enriched in hypoxic CSCC cell-derived exosomes and transferred to macrophages

Our previous study reported that TAMs in the hypoxic TME exert immunosuppressive effects while playing a tumour-supportive role [24]. Exosomes may play important roles in the phenotypic and functional changes in TAMs [25, 26]. Thus, we extracted exosomes from the supernatants of normoxic and hypoxic CSCC cells. A typical cup-shaped morphology was confirmed by TEM (Fig. 3A), and a size range of 30–150 nm was verified by NTA (Supplemental Fig. 4). Positive expression of exosome markers, including CD63, EpCAM and CD81, and negative expression of the exosome exclusion marker Calnexin were detected by western blotting (Fig. 3B). Furthermore, we found that ZEB1 was enriched in hypoxic CSCC cell-derived exosomes relative to cells and exosomes secreted by normoxic CSCC cells (Fig. 3B-C).

**Fig. 4:**
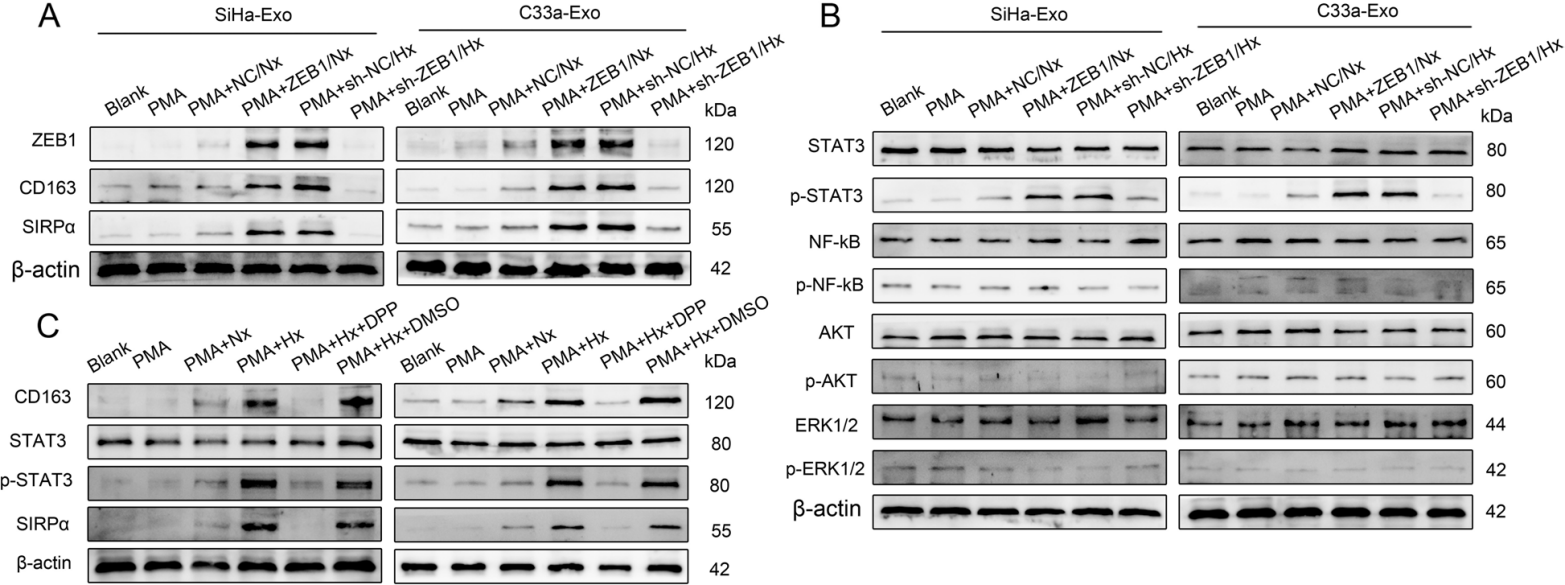
Hypoxia-induced exosomal ZEB1 promotes SIRPα^+^ TAM polarization through STAT3 signalling in vitro. **A** Western blot analysis showing the expression of ZEB1, CD163 and SIRPα in THP-1 macrophages under different conditions. **B** Related downstream signals for exosomal ZEB1 in THP-1 macrophages were evaluated by western blotting. **C** The protein levels of STAT3, p-STAT3, CD163 and SIRPα were measured in THP-1 macrophages incubated with 5,15-DPP (a specific inhibitor of STAT3 signalling) by western blotting. *, *P* < 0.05. Exo, exosome; Nx, normoxia; Hx, hypoxia

For the macrophage polarization experiment, THP-1 cells were first treated with 5 nM phorbol 12-myristate 13-acetate (PMA) for 6 h to acquire a macrophage-like phenotype. The resulting THP-1 macrophages were then polarized towards the M2 phenotype by incubation with tumour cell-derived factors; this model is the most widely used model for macrophage studies in vitro [27]. Purified exosomes were then labelled with the fluorescent membrane tracer PKH67 (green) and incubated with THP-1 macrophages. After 48 h of incubation, cells that internalized exosomes were stained with phalloidin (red) and DAPI (blue) for confocal microscopy evaluation. A punctate green fluorescence signal within the cytoplasm of recipient macrophages indicated the internalization of exosomes (Fig. 3D). A PBS-treated control group (bottom right corner) was established to rule out nonspecific internalization of exosomes into THP-1 cells. For in vitro treatment, 10 µg of exosomes resuspended in 100 µl of PBS was added to 1 × 10^5^ recipient cells. After internalization of normoxic and hypoxic CSCC-derived exosomes, THP-1 macrophages underwent morphological changes from a relatively round to an irregular shape (Fig. 3D), consistent with the TAM morphology observed in the TME. To confirm that hypoxic CSCC cell-derived ZEB1 can be transferred to THP-1 macrophages via exosomes, we measured ZEB1 levels in THP-1 macrophages pretreated with CSCC cell-derived exosomes. An increase in the intracellular level of ZEB1 was observed in recipient THP-1 macrophages following treatment with hypoxic CSCC cell-derived exosomes compared with normoxic CSCC cell-derived exosomes or PBS-treated exosomes (Fig. 3E). These data indicate that horizontal transfer of ZEB1 from hypoxic CSCC cells to macrophages can be mediated by exosomes.

### *Hypoxia-induced exosomal ZEB1 promotes SIRPα*^+^*TAM polarization through STAT3 signalling*

To investigate the role of exosomal ZEB1 in SIRPα^+^ TAM polarization, SiHa and C33a cells with stable ZEB1 overexpression or silencing were cultured under normoxic (Nx) or hypoxic (Hx) conditions. As shown in Supplemental Fig. 5, the ZEB1 level was significantly higher in exosomes derived from SiHa^ZEB1^/Nx, SiHa^sh−NC^/Hx, C33a^ZEB1^/Nx, and C33a^sh−NC^/Hx cells than in those derived from SiHa^NC^/Nx, SiHa^sh−ZEB1^/Hx, C33a^NC^/Nx, and C33a^sh−ZEB1^/Hx cells. Then, THP-1 macrophages were incubated with exosomes secreted by the above cells for 48 h. The results showed that exosomes with a high ZEB1 level dramatically promoted CD163 and SIRPα expression compared with those with a low ZEB1 level and also increased the ZEB1 expression level in recipient cells (Fig. 4A). Consistently, STAT3 phosphorylation in THP-1 macrophages was considerably increased after exposure to exosomes with a high ZEB1 level (Fig. 4B). 5,15-Diphenyl-porphine (5,15-DPP) was used to inhibit the nuclear translocation and accumulation of phosphorylated STAT3 [20]. After this inhibition, the induction of SIRPα^+^ TAM polarization was obviously suppressed (Fig. 4C), indicating a critical role for STAT3 activation in this M2-like TAM phenotypic switch.

**Fig. 5:**
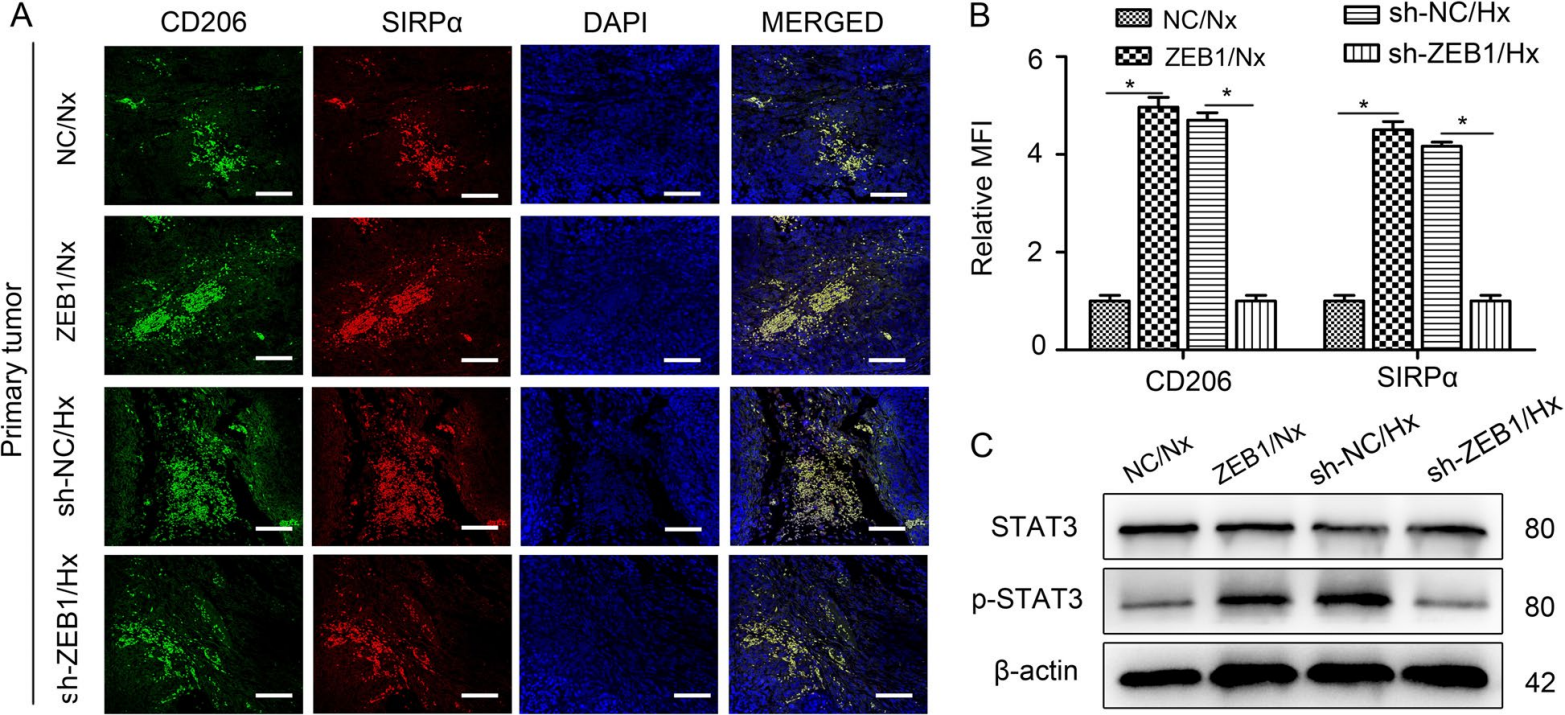
Hypoxia-induced exosomal ZEB1 promotes SIRPα^+^ TAM polarization through STAT3 signalling in vivo. An in vivo CSCC xenograft model was established in nude mice by inoculating SiHa cells (5 × 10^6^ cells/mouse) into the flank. When the tumour volume reached 50 mm^3^, exosomes (10 µg) secreted by SiHa^NC^/Nx, SiHa^ZEB1^/Nx, SiHa^sh−NC^/Hx, and SiHa^sh−ZEB1^/Hx cells were injected into the tumour centres (*n* = 3 mice/group) every other day for three weeks. The tumour volume was recorded every 4 days. After three weeks, the nude mice were euthanized using a barbiturate overdose, and the tumours were collected for analysis. **A** Representative images of STAT3 (purple), CD206 (red), SIRPα (green) and DAPI (blue) fluorescence staining in primary tumours from different treatment groups. Scale bar, 100 µm. **B** MFI analysis showing the relative expression of CD206 and SIRPα in the tumours described in (A). *, *P* < 0.05. MFI, mean fluorescence intensity. **C** M2-like TAMs were isolated from tumours and characterized ex vivo by western blot analysis of the levels of STAT3 and p-STAT3. Exo, exosome; Nx, normoxia; Hx, hypoxia

The effect of exosomal ZEB1 on SIRPα^+^ TAM polarization was further assessed in a CSCC xenograft model in vivo. We inoculated SiHa cells into the flanks of nude mice. When the tumour volume reached 50 mm^3^, exosomes secreted by SiHa^NC^/Nx, SiHa^ZEB1^/Nx, SiHa^sh−NC^/Hx, and SiHa^sh−ZEB1^/Hx cells were randomly injected into the centres of the xenograft tumours (*n* = 3 mice/group) every other day for three weeks. The tumour volume was recorded every 4 days. Treatment with exosomes derived from cells with ZEB1 overexpression significantly increased the size of the formed tumours, while the exosomes derived from ZEB1 knockdown cells had the opposite effect (both *P* < 0.05) (Supplemental Fig. 6). These results suggest that hypoxic CSCC cell-derived exosomal ZEB1 can promote tumour growth. After three weeks, the tumours were harvested for immunofluorescence staining and western blot analysis. Significantly higher levels of phosphorylated STAT3, CD206 (a TAM marker in mice) and SIRPα were present in the tumours treated with exosomes with a high ZEB1 level than in those treated with exosomes with a low ZEB1 level (Fig. 5A-B). M2-like TAMs were isolated from tumours and characterized ex vivo by western blot analysis of p-STAT3. The results showed that STAT3 phosphorylation was considerably increased after exposure to exosomes with a high ZEB1 level (Fig. 5C). Collectively, these data indicate that hypoxic CSCC cell-derived exosomal ZEB1 can promote SIRPα^+^ TAM polarization through STAT3 signalling in vitro and in vivo.

**Fig. 6:**
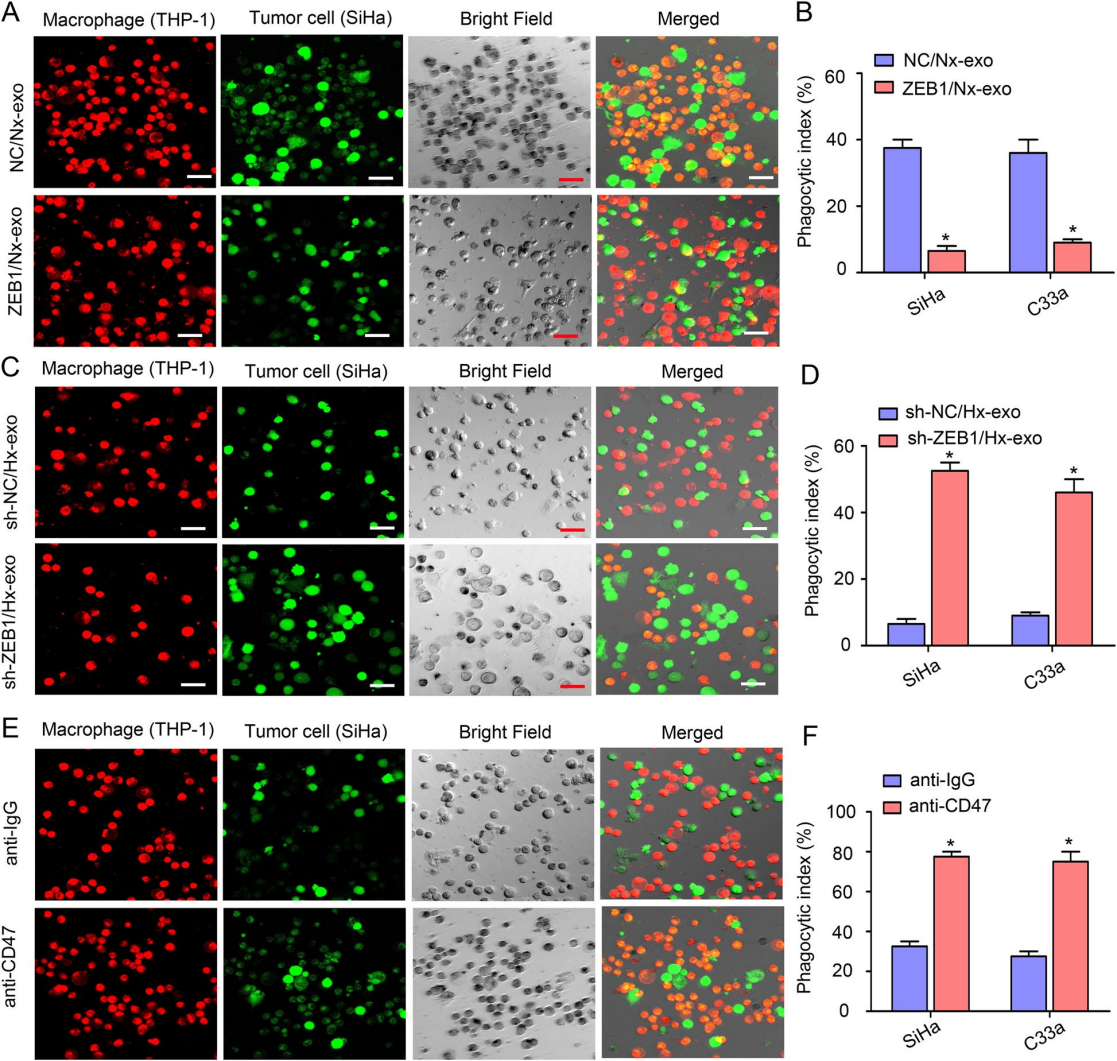
ZEB1 activity affects the phagocytosis of CSCC cells by regulating CD47 and SIRPα expression. DiI-labelled THP-1 macrophages were incubated with CFSE-labelled CSCC cells. After a 4-h incubation, phagocytic events were imaged under an inverted microscope. **A** THP-1 macrophages were incubated with exosomes secreted by normoxic SiHa cells transfected with the ZEB1 overexpression (ZEB1/Nx-exo) or control plasmid (NC/Nx-exo) before a phagocytosis assay was performed. **B** Statistical analysis showing the phagocytic index of the cells described in (A). **C** THP-1 macrophages were incubated with exosomes secreted by hypoxic SiHa cells infected with the ZEB1 knockdown virus (sh-ZEB1/Hx-exo) or the negative control virus (sh-NC/Hx-exo) before a phagocytosis assay was performed. **D** Statistical analysis showing the phagocytic index of the cells described in (C). **E** Phagocytosis assays were performed in the presence of an anti-CD47 blocking antibody or control IgG. **F** Statistical analysis showing the phagocytic index of the cells described in (E). Scale bar, 50 μm. *, *P* < 0.05. Exo, exosome; Nx, normoxia; Hx, hypoxia

### ZEB1 activity affects the phagocytosis of CSCC cells by regulating CD47 and SIRPα expression

We observed that ZEB1 increased CD47 and SIRPα expression in the hypoxic TME of CSCC, which led us to hypothesize that ZEB1 activity can affect the phagocytosis of CSCC cells. To investigate the effect of exosomal ZEB1 on macrophage phagocytosis, THP-1 macrophages were incubated with exosomes secreted by normoxic CSCC cells transfected with the ZEB1 overexpression plasmid (ZEB1/Nx-exo) or the control plasmid (NC/Nx-exo) and by hypoxic CSCC cells infected with the ZEB1 knockdown virus (sh-ZEB1/Hx-exo) or the negative control virus (sh-NC/Hx-exo) before a phagocytosis assay was performed. THP-1 macrophages were labelled with 5 μM DiI (red) in serum-free medium for 2 h, and CSCC cells were labelled with CFSE (green) and added to the macrophage cultures. After 4 h of incubation, phagocytic events were imaged under an inverted microscope. We observed a significant decrease in phagocytosis by THP-1 macrophages incubated with ZEB1/Nx-exo compared with NC/Nx-exo (Fig. 6A-B). Moreover, phagocytosis by macrophages treated with sh-ZEB1/Hx-exo was significantly increased compared with that by macrophages treated with sh-NC/Hx-exo (Fig. 6C-D). To confirm the involvement of CD47/SIRPα in the regulation of phagocytosis, we repeated the phagocytosis assay after blocking CD47 expression. Phagocytosis of CSCC cells by THP-1 macrophages was increased in the presence of the anti-CD47 blocking antibody (Fig. 6E-F). These results suggest that ZEB1 activity affects the phagocytosis of CSCC cells by regulating CD47 and SIRPα expression.

Collectively, our findings indicate that there is a ZEB1-dependent increase in the expression of CD47 when CSCC cells are exposed to hypoxia. In addition, exosomal ZEB1 derived from hypoxic CSCC cells promotes SIRPα^+^ TAM polarization via activation of the STAT3 signalling pathway and thus facilitates immune evasion by cancer cells.

## Discussion

Accumulating evidence indicates that in various human cancers, the CD47-SIRPα axis is required for escape from innate immune surveillance [28]. Blocking the interaction of CD47 with its receptor, SIRPα, on macrophages enables phagocytosis and inhibits tumour progression [29]. However, the molecular mediators and mechanisms regulating the expression of CD47 by cancer cells and SIRPα by macrophages remain poorly defined. In this study, we demonstrate that hypoxia, which is a critical microenvironmental stressor in advanced CSCC, induces ZEB1-dependent expression of CD47 and acquisition of the SIRPα^+^ TAM phenotype, leading to decreased phagocytosis of CSCC cells by macrophages, which could promote tumour progression and increase patient mortality.

Of interest, a deeper understanding of the regulation of CD47 expression on cancer cells at the molecular level has recently emerged [30]. For instance, Zhang et al. showed that the expression of CD47 in hypoxic breast cancer cells was linked to HIF-1-dependent transcription of CD47 [10]. However, we and others have reported that exposure of CSCC cells to hypoxia induces the activity of ZEB1 instead of the activity of HIF-1 [15, 31]. Hypoxia-induced ZEB1 activates the transcription of a large battery of genes encoding proteins that promote multiple steps in tumour progression, including tumour growth, stromal cell recruitment, extracellular matrix remodelling, and premetastatic niche formation [17]. Here, we found that ZEB1 also plays an important role in immune evasion by activating the expression of CD47, an antiphagocytic signal (Fig. 2 and Supplemental Fig. 3). Our in vitro data were supported by clinical data showing that CD47 expression correlated with ZEB1 expression in human CSCC patients and suggested a poor prognosis (Fig. 1). The results presented herein represent, to our knowledge, the first identification of a transcriptional regulator of CD47 expression in hypoxic CSCC cells, and further studies are required to determine whether ZEB1 cooperates with other transcription factors induced by the hypoxic TME.

Hypoxia-mediated tumour progression and treatment resistance are major clinical challenges in CSCC [32]. Exosomes released into the hypoxic TME may contribute to these challenges by transferring bioactive molecules between tumour cells and stromal cells [33]. Park and colleagues showed that hypoxia could induce tumour cell secretion of proteins with the potential to remodel the TME. More than 50% of these secreted proteins were found in exosomes [34]. However, whether oncogenic transcription factors are transferred via exosomes is unknown. In our previous study, we found that hypoxia increased the expression of ZEB1 in CSCC cells, which resulted in increased TAM infiltration [15]. Herein, we demonstrate that endogenous ZEB1 is detectable in exosomes and that hypoxia significantly increases the level of ZEB1 in exosomes derived from CSCC cells (Fig. 3). Exosomal ZEB1 may be internalized by recipient cells and translocate to the nucleus, where transcription factors are thought to function. Exosomal ZEB1 retains DNA-binding activity and is transcriptionally active in recipient cells after exosome uptake. In addition, we provide evidence that ZEB1 itself participates in exosome-mediated pro-metastatic effects on recipient cells, as exosome-mediated delivery of active forms of ZEB1 resulted in a TAM phenotypic switch via activation of STAT3 signalling (Figs. 4 and 5). A recent study reported that TAMs depend on ZEB1 to maintain a tumour-promoting phenotype and protumour functions, highlighting the importance of ZEB1 in TAMs [35]. Oncogene-induced STAT3 activation plays a central role in tumour progression by amplifying immunosuppressive activity in infiltrating TAMs [36]. In addition, TAMs educated by cancer cells can facilitate tumour migration and proliferation via a feedback loop [26]. Immunofluorescence analysis of CSCC tissues revealed that TAMs in hypoxic regions expressed a higher level of SIRPα than those in normoxic regions (Fig. 1). Consistent with this result, hypoxic CSCC cells also induced dynamic expression of SIRPα in macrophages, accompanied by an alteration in TAM polarization in vitro, suggesting that SIRPα expression correlates with the TAM phenotypic switch (Fig. 4). SIRPα is a cell surface glycoprotein considered an inhibitory molecule due to the ITIM domains in its intracellular region [37]. Tyrosine phosphorylation of SIRPα is increased in macrophages cocultured with tumour cells, resulting in SHP2 recruitment to the cell surface [38]. Moreover, it has been reported that SIRPα is required for T and natural killer (NK) cell homeostasis in vivo, indicating an important role for SIRPα in immunomodulation [39]. However, further investigation is required to determine the role of SIRPα in the cross-talk of TAMs with other immune cells in the TME.

The CD47-SIRPα interaction serves as a “don’t eat me” and a “self-recognition” signal [7]. Blocking the CD47-SIRPα interaction has been shown to promote the phagocytosis of cancer cells by macrophages (Fig. 6). In our study, the effects of ZEB1 and CD47-SIRPα were similar, but the latest studies show that they also have complementary and synergistic effects. For example, ZEB1 is known to inhibit effector T cells through a counterregulatory network in the TME [40], suggesting that combination treatment with a ZEB1 antagonist may improve the response to CD47-SIRPα blockade by disinhibiting T-cell-mediated antitumour immunity. Another interesting observation is that blocking the CD47-SIRPα pathway can effectively promote TAM repolarization. Zhang et al. found that anti-CD47 treatment alone could shift the polarization of macrophages towards the M1 phenotype [41]. Chen et al. developed an in situ-formed fibrin gel with calcium carbonate nanoparticles (NPs) loaded with anti-CD47 antibodies, which allowed repolarization of M2 macrophages into M1 macrophages and promoted phagocytosis by macrophages [42]. These studies suggest that the combined effect of ZEB1 and CD47 may be even more powerful. The immune checkpoint-based cancer immunotherapy revolution is in its infancy, and combination cancer immunotherapy is an area of great interest in the field [43]. We need to design safe, robust, synergistic and rational combination immunotherapies to maximize the benefit to a larger portion of CSCC patients [44]. Here, we describe a new mechanism in which hypoxia-induced ZEB1 is crucial for CD47 expression and SIRPα^+^ TAM polarization, which may be helpful for designing new antitumour drugs. For example, reprogramming TAMs using a ZEB1 antagonist and blocking the CD47-SIRPα pathway are state-of-the-art “normalization cancer immunotherapy” strategies that can synergistically restore the antitumour properties of immune cells in the TME. In addition, according to the latest research, inactivation of ZEB1 may offer alternative approaches for cancer therapy with minimal side effects [45]. Therefore, ZEB1-targeted therapy in combination with CD47-SIRPα checkpoint immunotherapy may yield a potent and superior antitumour effect, thus effectively suppressing CSCC progression.

## Conclusions

In summary, our findings provide new insight into the importance of hypoxia-induced ZEB1 in immune evasion and suggest that there is fine-tuned collaborative action between ZEB1 and the CD47-SIRPα axis. Based on our results, we propose a combination regimen using a ZEB1 antagonist as an adjuvant along with simultaneous blockade of the CD47-SIRPα immune checkpoint, which may improve the outcomes of the majority of patients with CSCC in part by disinhibiting innate immunity.

### Supplementary Information

**Additional file 1:** **Supplemental Table 1.** ZEB1-overexpression sequence.

**Additional file 2:** **Supplemental Table 2.** CD47 (NM_001777-promoter).

**Additional file 3:** **Supplemental Table 3.** (Related to Figure 1). Clinical and pathological characteristics of CSCC samples used in this study.

**Additional file 4:** **Supplemental Figure 1.** Hypoxia increases CD47 expression in CSCC cells. FCM analysis showing the expression of CD47 in normoxic/hypoxic SiHa and C33a cells. *, *P*<0.05. MFI, mean fluorescence intensity.

**Additional file 5:** **Supplemental Figure 2.** Hypoxia promotes EMT in CSCC cells.

**Additional file 6:** **Supplemental Figure 3.** Hypoxia-induced ZEB1 increases CD47 expression in C33a cells.

**Additional file 7:** **Supplemental Figure 4.** Exosome size identification by nanoparticle tracking analysis (NTA). Exosomes secreted by normoxic and hypoxic CSCC cells (SiHa and C33a) were analysed by the NanoSight system.

**Additional file 8:** **Supplemental Figure 5.** ZEB1 expression levels in exosomes derived from CSCC cells.

**Additional file 9:** **Supplemental Figure 6.** Growth curve of CSCC xenograft model* in vivo*. *, *P*<0.05. Nx, normoxia; Hx, hypoxia; CSCC, cervical squamous cell carcinoma.

**Additional file 10:**

## Data Availability

All data generated or analyzed during this study are included in this published article.

## Abbreviations

CSCC: Cervical squamous cell carcinoma
TME: Tumour microenvironment
TAMs: Tumour-associated macrophages
SIRPα: Signal regulatory protein α
CAIX: Carbonic anhydrase IX
CM: Conditioned medium
TEM: Transmission electron microscopy
PBS: Phosphate-buffered saline
EMT: Epithelial-to-mesenchymal transition
PMA: Phorbol 12-myristate 13-acetate
Hx: Hypoxia
Nx: Normoxia
Exo: Exosome
