# Supplementary material for: Hypoxia-induced ZEB1 promotes cervical cancer immune evasion by strengthening the CD47-SIRPα axis
Source: Cell Commun Signal. 2024 Jan 5;22:15. doi: 10.1186/s12964-023-01450-4 (PMC10768116; doi:10.1186/s12964-023-01450-4)
Supplement: Supplementary file 1 — Additional file 1: Supplemental Table 1. ZEB1-overexpression sequence. [file 12964_2023_1450_MOESM1_ESM.docx]

**Supplemental Table 1**

**ZEB1-overexpression sequence:**

>EX-Z5750-M02 ORF sequence

ATGAAAGTTACAAATTATAATACTGTGGTAGAAACAAATTCAGATTCAGATGATGAAGACAAACTGCATATTGTGGAAGAAGAAAGTGTTACAGATGCAGCTGACTGTGAAGGTGTACCAGAGGATGACCTGCCAACAGACCAGACAGTGTTACCAGGGAGGAGCAGTGAAAGAGAAGGGAATGCTAAGAACTGCTGGGAGGATGACACAGGAAAGGAAGGGCAAGAAATCCTGGGGCCTGAAGCTCAGGCAGATGAAGCAGGATGTACAGTAAAAGATGATGAATGCGAGTCAGATGCAGAAAATGAGCAAAACCATGATCCTAATGTTGAAGAGTTTCTACAACAACAAGACACTGCTGTCATTTTTCCTGAGGCACCTGAAGAGGACCAGAGGCAGGGCACACCAGAAGCCAGTGGTCATGATGAAAATGGAACACCAGATGCATTTTCACAATTACTCACCTGTCCATATTGTGATAGAGGCTATAAACGCTTTACCTCTCTGAAAGAACACATTAAATATCGTCATGAAAAGAATGAAGATAACTTTAGTTGCTCCCTGTGCAGTTACACCTTTGCATACAGAACCCAACTTGAACGTCACATGACATCACATAAATCAGGAAGAGATCAAAGACATGTGACGCAGTCTGGGTGTAATCGTAAATTCAAATGCACTGAGTGTGGAAAAGCTTTCAAATACAAACATCACCTAAAAGAGCACTTAAGAATTCACAGTGGAGAGAAGCCATATGAATGCCCAAACTGCAAGAAACGCTTTTCCCATTCTGGCTCCTATAGCTCACACATAAGCAGTAAGAAATGTATCAGCTTGATACCTGTGAATGGGCGACCAAGAACAGGACTCAAGACATCTCAGTGTTCTTCACCGTCTCTTTCAGCATCACCAGGCAGTCCCACACGACCACAGATACGGCAAAAGATAGAGAATAAACCCCTTCAAGAACAACTTTCTGTTAACCAAATTAAAACTGAACCTGTGGATTATGAATTCAAACCCATAGTGGTTGCTTCAGGAATCAACTGTTCAACCCCTTTACAAAATGGGGTTTTCACTGGTGGTGGCCCATTACAGGCAACCAGTTCTCCTCAGGGCATGGTGCAAGCTGTTGTTCTGCCAACAGTTGGTTTGGTGTCTCCCATAAGTATCAATTTAAGTGATATTCAGAATGTACTTAAAGTGGCGGTAGATGGTAATGTAATAAGGCAAGTGTTGGAGAATAATCAAGCCAATCTTGCATCCAAAGAACAAGAAAC

AATCAATGCTTCACCCATACAACAAGGTGGCCATTCTGTTATTTCAGCCATCAGTCTTCCTTTGGTTGATCAAGATGGAACAACCAAAATTATCATCAACTACAGTCTTGAGCAGCCTAGCCAACTTCAAGTTGTTCCTCAAAATTTAAAAAAAGAAAATCCAGTCGCTACAAACAGTTGTAAAAGTGAAAAGTTACCAGAAGATCTTACTGTTAAGTCTGAGAAGGACAAAAGCTTTGAAGGGGGGGTGAATGATAGCACTTGTCTTCTGTGTGATGATTGTCCAGGAGATATTAATGCACTTCCAGAATTAAAGCACTATGACCTAAAGCAGCCTACTCAGCCTCCTCCACTCCCTGCAGCAGAAGCTGAGAAGCCTGAGTCCTCTGTTTCATCAGCTACTGGAGATGGCAATTTGTCTCCTAGTCAGCCACCTTTAAAGAACCTCTTGTCTCTCCTAAAAGCATATTATGCTTTGAATGCACAACCAAGTGCAGAAGAGCTCTCAAAAATTGCTGATTCAGTAAACCTACCACTGGATGTAGTAAAAAAGTGGTTTGAAAAGATGCAAGCTGGACAGATTTCAGTGCAGTCTTCTGAACCATCTTCTCCTGAACCAGGCAAAGTAAATATCCCTGCCAAGAACAATGATCAGCCTCAATCTGCAAATGCAAATGAACCCCAGGACAGCACAGTAAATCTACAAAGTCCTTTGAAGATGACTAACTCCCCAGTTTTACCAGTGGGATCAACCACCAATGGTTCCAGAAGTAGTACACCATCCCCATCACCTCTAAACCTTTCCTCATCCAGAAATACACAGGGTTACTTGTACACAGCTGAGGGTGCACAAGAAGAGCCACAAGTAGAACCTCTTGATCTTTCACTACCAAAGCAACAGGGAGAATTATTAGAAAGGTCAACTATCACTAGTGTTTACCAGAACAGTGTTTATTCTGTCCAGGAAGAACCCTTGAACTTGTCTTGCGCAAAAAAGGAGCCACAAAAGGACAGTTGTGTTACAGACTCAGAACCAGTTGTAAATGTAATCCCACCAAGTGCCAACCCCATAAATATCGCTATACCTACAGTCACTGCCCAGTTACCCACAATCGTGGCCATTGCTGACCAGAACAGTGTTCCATGCTTAAGAGCGCTAGCTGCCAATAAGCAAACGATTCTGATTCCCCAGGTGGCATACACCTACTCAACTACGGTCAGCCCTGCAGTCCAAGAACCACCCTTGAAAGTGATCCAGCCAAATGGAAATCAGGATGAAAGACAAGATACTAGCTCAGAAGGAGTATCAAATGTAGAGGATCAGAATGACTCTGATTCTACACCGCCCAAAAAGAAAATGCGGAAGACAGAAAATGGAATGTATGCTTGTGATTTGTGTGACAAGATATTCCAAAAGAGTAGTTCATTATTGAGACATAAATATGAACACACAGGTAAAAGACCTCATGAGTGTGGAATCTGTAAAAAGGCATTTAAACACAAACATCATTTGATTGAAC

ACATGCGATTACATTCTGGAGAAAAGCCCTATCAATGTGACAAATGTGGAAAGCGCTTCTCACACTCTGGGTCTTATTCTCAACACATGAATCATCGCTACTCCTACTGTAAGAGAGAAGCGGAAGAACGTGACAGCACAGAGCAGGAAGAGGCAGGGCCTGAAATCCTCTCGAATGAGCACGTGGGTGCCAGGGCGTCTCCCTCACAGGGCGACTCGGACGAGAGAGAGAGTTTGACAAGGGAAGAGGATGAAGACAGTGAAAAAGAGGAAGAGGAGGAGGATAAAGAGATGGAAGAATTGCAGGAAGAAAAAGAATGTGAAAAACCACAAGGGGATGAGGAAGAGGAGGAGGAGGAGGAAGAAGTGGAAGAAGAAGAGGTAGAAGAGGCAGAGAATGAGGGAGAAGAAGCAAAAACTGAAGGTCTGATGAAGGATGACAGGGCTGAAAGTCAAGCAAGCAGCTTAGGACAAAAAGTAGGCGAGAGTAGTGAGCAAGTGTCTGAAGAAAAGACAAATGAAGCC

**NC sequence:**

>EX-NEG-M02-B

AACCCAGCTTTCTTGTACAAAGTGGTTGATCGCGTGCATGCGACGTCATAGCTCTCTCCCTATAGTGAGTCGTATTATAAGCTAGGCACTGGCCGTCGTTTTACAACGTCGTGACTGGGAAAACTGCTAGCTTGGGATCTTTGTGAAGGAACCTTACTTCTGTGGTGTGACATAATTGGACAAACTACCTACAGAGATTTAAAGCTCTAAGGTAAATATAAAATTTTTAAGTGTATAATGTGTTAAACTAGCTGCATATGCTTGCTGCTTGAGAGTTTTGCTTACTGAGTATGATTTATGAAAATATTATACACAGGAGCTAGTGATTCTAATTGTTTGTGTATTTTAGATTCACAGTCCCAAGGCTCATTTCAGGCCCCTCAGTCCTCACAGTCTGTTCATGATCATAATCAGCCATACCACATTTGTAGAGGTTTTACTTGCTTTAAAAAACCTCCCACACCTCCCCCTGAACCTGAAACATAAAATGAATGCAATTGTTGTTGTTAACTTGTTTATTGCAGCTTATAATGGTTACAAATAAAGCAATAGCATCACAAATTTCACAAATAAAGCATTTTTTTCACTGCATTCTAGTTGTGGTTTGTCCAAACTCATCAATGTATCTTATCATGTCTGGATCGATCCTGCATTAATGAATCGGCCAACGCGCGGGGAGAGGCGGTTTGCGTATTGGCTGGCGTAATAGCGAAGAGGCCCGCACCGATCGCCCTTCCCAACAGTTGCGCAGCCTGAATGGCGAATGGGACGCGCCCTGTAGCGGCGCATTAAGCGCGGCGGGTGTGGTGGTTACGCGCAGCGTGACCGCTACACTTGCCAGCGCCCTAGCGCCCGCTCCTTTCGCTTTCTTCCCTTCCTTTCTCGCCACGTTCGCCGGCTTTCCCCGTCAAGCTCTAAATCGGGGGCTCCCTTTAGGGTTCCGATTTAGTGCTTTACGGCACCTCGACCCCAAAAAACTTGATTAGGGTGATGGTTCACGTAGTGGGCCATCGCCCTGATAGACGGTTTTTCGCCCTTTGACGTTGGAGTCCACGTTCTTTAATAGTGGACTCTTGTTCCAAACTGGAACAACACTCAACCCTATCTCGGTCTATTCTTTTGATTTATAAGGGATTTTGCCGATTTCGGCCTATTGGTTAAAAAATGAGCTGATTTAACAAATATTTAACGCGAATTTTAACAAAATATTAACGTTTACAATTTCGCCTGATGCGGTATTTTCTCCTTACGCATCTGTGCGGTATTTCACACCGCATACGCGGATCTGCGCAGCACCATGGCCTGAAATAACCTCTGAAAGAGGAACTTGGTTAGGAACCTTCTGAGGCGGAAAGAACCAGCTGTGGAATGTGTGTCAGTTAGGGTGTGGAAAGTCCCCAGGCTCCCCAGCAGGCAGAAGTATGCAAAGCATGCATCTCAATTAGTCAGCAACCAGGTGTGGAAAGTCCCCAGGCTCCCCAGCAGGCAGAAGTATGCAAAGCATGCATCTCAATTAGTCAGCAACCATAGTCCCGCCCCTAACTCCGCCCATCCCGCCCTAACTCCGCCCAGTTCCGCCCATTCTCCGCCCCATGGCTGACTAATTTTTTTTATTTATGCAGAGGCCGAGGCCGCCTCGGCCTCTGAGCTATTCCAGAAGTAGTGAGGAGGCTTTTTTGGAGGCCTAGGCTTTTGCAAAAAGCTTGATTCTTCTGACACAACAGTCTCGAACTTAAGGCTAGAGCCACCATGATTGAACAAGATGGATTGCACGCAGGTTCTCCGGCCGCTTGGGTGGAGAGGCTATTCGGCTATGACTGGGCACAACAGACAATCGGCTGCTCTGATGCCGCCGTGTTCCGGCTGTCAGCGCAGGGGCGCCCGGTTCTTTTTGTCAAGACCGACCTGTCCGGTGCCCTGAATGAACTGCAGGACGAGGCAGCGCGGCTATCGTGGCTGGCCACGACGGGCGTTCCTTGCGCAGCTGTGCTCGACGTTGTCACTGAAGCGGGAAGGGACTGGCTGCTATTGGGCGAAGTGCCGGGGCAGGATCTCCTGTCATCTCACCTTGCTCCTGCCGAGAAAGTATCCATCATGGCTGATGCAATGCGGCGGCTGCATACGCTTGATCCGGCTACCTGCCCATTCGACCACCAAGCGAAACATCGCATCGAGCGAGCACGTACTCGGATGGAAGCCGGTCTTGTCGATCAGGATGATCTGGACGAAGAGCATCAGGGGCTCGCGCCAGCCGAACTGTTCGCCAGGCTCAAGGCGCGCATGCCCGACGGCGAGGATCTCGTCGTGACCCATGGCGATGCCTGCTTGCCGAATATCATGGTGGAAAATGGCCGCTTTTCTGGATTCATCGACTGTGGCCGGCTGGGTGTGGCGGACCGCTATCAGGACATAGCGTTGGCTACCCGTGATATTGCTGAAGAGCTTGGCGGCGAATGGGCTGACCGCTTCCTCGTGCTTTACGGTATCGCCGCTCCCGATTCGCAGCGCATCGCCTTCTATCGCCTTCTTGACGAGTTCTTCTGAGCGGGACTCTGGGGTTcgcGAAATGACCGACCAAGCGACGCCCAACCTGCCATCACGATGGCCGCAATAAAATATCTTTATTTTCATTACATCTGTGTGTTGGTTTTTTGTGTGAATCGATAGCGATAAGGATCCGCGTATGGTGCACTCTCAGTACAATCTGCTCTGATGCCGCATAGTTAAGCCAGCCCCGACACCCGCCAACACCCGCTGAC

GCGCCCTGACGGGCTTGTCTGCTCCCGGCATCCGCTTACAGACAAGCTGTGACCGTCTCCGGGAGCTGCATGTGTCAGAGGTTTTCACCGTCATCACCGAAACGCGCGAGACGAAAGGGCCTCGTGATACGCCTATTTTTATAGGTTAATGTCATGATAATAATGGTTTCTTAGACGTCAGGTGGCACTTTTCGGGGAAATGTGCGCGGAACCCCTATTTGTTTATTTTTCTAAATACATTCAAATATGTATCCGCTCATGAGACAATAACCCTGATAAATGCTTCAATAATATTGAAAAAGGAAGAGTATGAGTATTCAACATTTCCGTGTCGCCCTTATTCCCTTTTTTGCGGCATTTTGCCTTCCTGTTTTTGCTCACCCAGAAACGCTGGTGAAAGTAAAAGATGCTGAAGATCAGTTGGGTGCACGAGTGGGTTACATCGAACTGGATCTCAACAGCGGTAAGATCCTTGAGAGTTTTCGCCCCGAAGACCGTTTTCCAATGATGAGCACTTTTAAAGTTCTGCTATGTGGCGCGGTATTATCCCGTATTGACGCCGGGCAAGAGCAACTCGGTCGCCGCATACACTATTCTCAGAATGACTTGGTTGAGTACTCACCAGTCACAGAAAAGCATCTTACGGATGGCATGACAGTAAGAGAATTATGCAGTGCTGCCATAACCATGAGTGATAACACTGCGGCCAACTTACTTCTGACAACGATCGGAGGACCGAAGGAGCTAACCGCTTTTTTGCACAACATGGGGGATCATGTAACTCGCCTTGATCGTTGGGAACCGGAGCTGAATGAAGCCATACCAAACGACGAGCGTGACACCACGATGCCTGTAGCAATGGCAACAACGTTGCGCAAACTATTAACTGGCGAACTACTTACTCTAGCTTCCCGGCAACAATTAATAGACTGGATGGAGGCGGATAAAGTTGCAGGACCACTTCTGCGCTCGGCCCTTCCGGCTGGCTGGTTTATTGCTGATAAATCTGGAGCCGGTGAGCGTGGGTCTCGCGGTATCATTGCAGCACTGGGGCCAGATGGTAAGCCCTCCCGTATCGTAGTTATCTACACGACGGGGAGTCAGGCAACTATGGATGAACGAAATAGACAGATCGCTGAGATAGGTGCCTCACTGATTAAGCATTGGTAACTGTCAGACCAAGTTTACTCATATATACTTTAGATTGATTTAAAACTTCATTTTTAATTTAAAAGGATCTAGGTGAAGATCCTTTTTGATAATCTCATGACCAAAATCCCTTAACGTGAGTTTTCGTTCCACTGAGCGTCAGACCCCGTAGAAAAGATCAAAGGATCTTCTTGAGATCCTTTTTTTCTGCGCGTAATCTGCTGCTTGCAAACAAAAAAACCACCGCTACCAGCGGTGGTTTGTTTGCCGGATCAAGAGCTACCAACTCTTTTTCCGAAGGTAACTGGCTTCAGCAGAGCGCAGATACCAAATACTGTCCTTCTAGTGTAGCCGTAGTTAGGCCACCACTTCAAGAACTCTGTAGCACCGCCTACATACCTCGCTCTGCTAATCCTGTTACCAGTGGCTGCTGCCAGTGGCGATAAGTCGTGTCTTACCGGGTTGGACTCAAGACGATAGTTACCGGATAAGGCGCAGCGGTCGGGCTGAACGGGGGGTTCGTGCACACAGCCCAGCTTGGAGCGAACGACCTACACCGAACTGAGATACCTACAGCGTGAGCATTGAGAAAGCGCCACGCTTCCCGAAGGGAGAAAGGCGGACAGGTATCCGGTAAGCGGCAGGGTCGGAACAGGAGAGCGCACGAGGGAGCTTCCAGGGGGAAACGCCTGGTATCTTTATAGTCCTGTCGGGTTTCGCCACCTCTGACTTGAGCGTCGATTTTTGTGATGCTCGTCAGGGGGGCGGAGCCTATGGAAAAACGCCAGCAACGCGGCCTTTTTACGGTTCCTGGCCTTTTGCTGGCCTTTTGCTCACATGTTCTTTCCTGCGTTATCCCCTGATTCTGTGGATAACCGTATTACCGCCTTTGAGTGAGCTGATACCGCTCGCCGCAGCCGAACGACCGAGCGCAGCGAGTCAGTGAGCGAGGAAGCGGAAGAGCGCCCAATACGCAAACCGCCTCTCCCCGCGCGTTGGCCGATTCATTAATGCAGAGCTTGCAATTCGCGCGTTTTTCAATATTATTGAAGCATTTATCAGGGTTATTGTCTCATGAGCGGATACATATTTGAATGTATTTAGAAAAATAAACAAATAGGGGTTCCGCGCACATTTCCCCGAAAAGTGCCACCTGACGTCTAAGAAACCATTATTATCATGACATTAACCTATAAAAATAGGCGTAGTACGAGGCCCTTTCACTCATTAGATGCATGTCGTTACATAACT

TACGGTAAATGGCCCGCCTGGCTGACCGCCCAACGACCCCCGCCCATTGACGTCAATAATGACGTATGTTCCCATAGTAACGCCAATAGGGACTTTCCATTGACGTCAATGGGTGGAGTATTTACGGTAAACTGCCCACTTGGCAGTACATCAAGTGTATCATATGCCAAGTACGCCCCCTATTGACGTCAATGACGGTAAATGGCCCGCCTGGCATTATGCCCAGTACATGACCTTATGGGACTTTCCTACTTGGCAGTACATCTACGTATTAGTCATCGCTATTACCATGGTGATGCGGTTTTGGCAGTACATCAATGGGCGTGGATAGCGGTTTGACTCACGGGGATTTCCAAGTCTCCACCCCATTGACGTCAATGGGAGTTTGTTTTGGCACCAAAATCAACGGGACTTTCCAAAATGTCGTAACAACTCCGCCCCATTGACGCAAATGGGCGGTAGGCGTGTACGGTGGGAGGTCTATATAAGCAGAGCTCGTTTAGTGAACCGTCAGATCGCCTGGAGACGCCATCCACGCTGTTTTGACCTCCATAGAAGACACCGGGACCGATCCAGCCTCCGGACTCTAGCCTAgatcTTCGAAGGAATTCGGTACCATGTAGCTCGAGTGCGGCCGC

**ZEB1-knockdown sequence (sh-ZEB1) :**

GAACCAGTTGTAAATGTAA

**sh-NC sequence:**

TTCTCCGAACGTGTCACGT
