# Supplementary material for: Hypoxia-induced ZEB1 promotes cervical cancer immune evasion by strengthening the CD47-SIRPα axis
Source: Cell Commun Signal. 2024 Jan 5;22:15. doi: 10.1186/s12964-023-01450-4 (PMC10768116; doi:10.1186/s12964-023-01450-4)
Supplement: Supplementary file 2 — Additional file 2: Supplemental Table 2. CD47 (NM_001777-promoter). [file 12964_2023_1450_MOESM2_ESM.docx]

**Supplemental Table 2**

**CD47 (NM_001777-promoter):**

AAATACTGAAAAGAGCTTAAGGTTATCTCAGAGAGAGGATAATCAAACCTAGGCAGGCTGCTTTGGACTGTCTCTCCTGGGATATGCCTGCTTTTGCCCCACCACCAAAACATACCCCAGCTAGATTCCCAAGAGCAGCAGTGGTCACCCAGGTGGCCTCTCATTCCATTTTCTGCACAAAAAGACGTTAAGCATATAGCTCAGTAGCTCCCAGTGTAAAACAGAACATCACCCGATCTCCACCTCTGAAGGTTGGGGACAGTCCTTTCTAGTCACTCTTCAGCTTTAGGGGGGTTGGTTGGTATCAGGGACAGCTGACAGCCTTATTTAGCCAACCAGCTGCTCTGTGATACACAATGTCCCTAATGCTCAAAGTCTGGTGGATGATTCTTTTGTATCCACATAAGCAGCAGTTGGAGGAGAATCGATGAGTCCCTTTGGTTGCCTCGGGGTGTCAAGGCTGCCATTCAAATCATTACCACCATAATTATTAACCATTGTTTTAGTGAGATGTCTGCCAGGCAATTGTTTTTATTTTTCAATCATTAAAAAAGCAATCAAATTTCACTAGAGCAATGCCTGCCTCACGCCGCATCAACACATTTATTAAACCCCTTCTGTTTGCCGAGCTCAATGGAAAGTCTTGGAGGAGGGAATACTTAAAATGCTCTCGGATTTAAAAGAATTGATAATGCTGTGGGGAAGAGGTTCACACAAAAAAGCAATTACAGGAAGAAGCTCGATAGTATACAATACACTGGTACAGAGTGTCATAGACAGTGCCACTTTCATACGCTGGATTTTATTTCTGTGGCAATGGGATGCTTGGGAGGAGCCGCACTGTGTAGAGGATTTGGAGAAGTGGGGTATTGTGGTGGGAAATTGCTTTCTTTCCCAGGAGGTAGGAGGAAAACAATCAAGGAGGTGGACAGGATGTGCACTCCATTAGAGCAGCCACCAGAGCCTGACTTTTTGATAAGAGAGTACATCAGTTAGGATAACGGTTAAAAGTATCTTTAAAAGACTTTTGCTTCAGGATGAATGATGTGGCCTGTGTGATTCAGCGATAAATTCAAAAGCCTTGTCCCTATTGTGGCTTGCGGCCACATTTCGAACCCATTTTTCAAGCATGTTAAACCCAAGCGCAGCGCAGAGGGCTGCACATGGGGCAGTCACAAACCAAGCTCAATAACCTTGCTGGTGGGGATGTGTTGGATACGCTGCTAATGCCTGTTTGCGACAATGCTCGCTAGTCCCGGTGGTGGCGGTGTTCACAGGTAACAATGTTTACCACCGTGAATGGAACTTGTTTGATTAACCCTGATCAGAGGATGAAAACACTAAAGAACCAAGTGAGAAAGAGGGAAGAGAACCGCATAGGGAAGAGCAGAGCGAGTAGACGAGCCGAACGCAGAGCCCGCGAGGGGCGAGTGGAAGCTCCCTGCGGGCAGGTACCCGACCACCGCCCTGCCCTGGGCGTGGCGGCCTCGGGCTCAGGGACCGCTTCGGCGCTAGACGGCCGCGTCCGGAGGAAACGGGCGCTGGTGAAAGCCTAGGTGTCCTGGTCCACGCGCGCAGCCGGACGTCGGGTCCAGGGAGAGACGCGGGCTGGGGCGGGACGGGACCCGGCCCCTGAAGCGCGAGGGTGGGAGTGAAAGCAAAGAGGAGAAAAGTAGAGAGAGAGGACAGTGGGGCCCAGCGCCGCGCGAAAGGCAGGAACCGACCCGCGGACAGGAACGGGTGCAATGAGGTCCCCGGCGAGCGTGGGAACACAGGGTTCAGCCTCCTGCGGCGGGCGAGCACGCGGACCCCAGGGGCGGGCGGGTGCGACAGGACGTGACCTGGAAGCGCGGCGCGTGCCACCGCCCTGGAGCAGGCATCCGGCCTCCGTGGAGCGGGCAGGCGGGCCCCGGGTCTGGAGCCTGCGACTGGGGAGGGCGCCGCGTCAACAGCAGCGGTTGCGGGGCGGGGCCGAGTGCGCGTGCGCGGCTCTCGCGGGCGGGGAGCAGGCGGGGGAGCGGGCGGGAAGCAGTGGGAG
