# Supplementary material for: Hypoxia-induced ZEB1 promotes cervical cancer immune evasion by strengthening the CD47-SIRPα axis
Source: Cell Commun Signal. 2024 Jan 5;22:15. doi: 10.1186/s12964-023-01450-4 (PMC10768116; doi:10.1186/s12964-023-01450-4)
Supplement: Supplementary file 3 — Additional file 3: Supplemental Table 3. (Related to Figure 1). Clinical and pathological characteristics of CSCC samples used in this study. [file 12964_2023_1450_MOESM3_ESM.docx]

Supplemental Table 3 (Related to Figure 1). Clinical and pathological characteristics of CSCC samples used in this study.

| **Case No.** | **ID** | **Diagnosis** | **Differentiation** | **Histology** | **Lymph node metastasis** | **FIGO stage** |
| --- | --- | --- | --- | --- | --- | --- |
| 1 | PA1302186 | cevical cancer | Low | Squamous cell carcinoma | YES | IB1 |
| 2 | PA1218175 | cevical cancer | Middle | Squamous cell carcinoma | YES | IIB |
| 3 | PA1203142 | cevical cancer | Middle | Squamous cell carcinoma | NO | IB1 |
| 4 | PA1206296 | cevical cancer | Middle | Squamous cell carcinoma | NO | IIB |
| 5 | PA1208609 | cevical cancer | Middle | Squamous cell carcinoma | NO | IIB1 |
| 6 | PA1305041 | cevical cancer | Low | Squamous cell carcinoma | NO | IB |
| 7 | PA1325890 | cevical cancer | High | Squamous cell carcinoma | NO | IIA1 |
| 8 | PA1414782 | cevical cancer | Low | Squamous cell carcinoma | NO | IIB |
| 9 | PA1305096 | cevical cancer | Low | Squamous cell carcinoma | NO | IB1 |
| 10 | PA1307614 | cevical cancer | Middle | Squamous cell carcinoma | NO | IB1 |
| 11 | PA1302735 | cevical cancer | Low | Squamous cell carcinoma | YES | IIB |
| 12 | PA1306652 | cevical cancer | Low | Squamous cell carcinoma | YES | IIA1 |
| 13 | PA1206070 | cevical cancer | Low | Squamous cell carcinoma | YES | IIA |
| 14 | PA1213702 | cevical cancer | Low | Squamous cell carcinoma | YES | IIB |
| 15 | PA1405501 | cevical cancer | Middle | Squamous cell carcinoma | YES | IIB |
| 16 | PA1210379 | cevical cancer | High | Squamous cell carcinoma | NO | IB2 |
| 17 | PA1304271 | cevical cancer | Middle | Squamous cell carcinoma | NO | IB2 |
| 18 | PA1402980 | cevical cancer | High | Squamous cell carcinoma | NO | IB |
| 19 | PA1426739 | cevical cancer | Low | Squamous cell carcinoma | NO | IB1 |
| 20 | PA1426861 | cevical cancer | Middle | Squamous cell carcinoma | NO | IIA1 |
