# Supplementary figures and images for: Hypoxia-induced ZEB1 promotes cervical cancer immune evasion by strengthening the CD47-SIRPα axis
Source: Cell Commun Signal. 2024 Jan 5;22:15. doi: 10.1186/s12964-023-01450-4 (PMC10768116; doi:10.1186/s12964-023-01450-4)

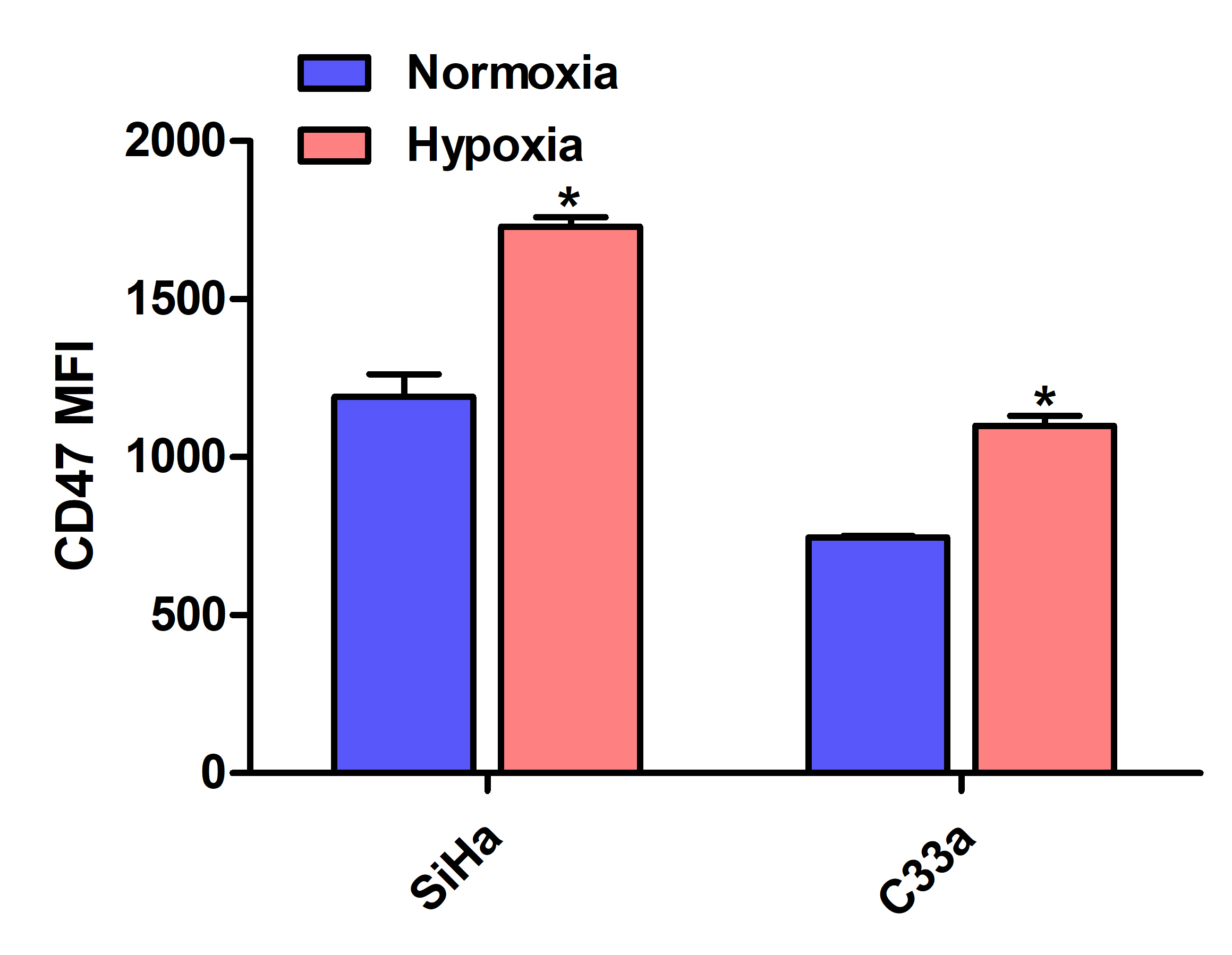

Supplement: Supplementary file 4 — Additional file 4: Supplemental Figure 1. Hypoxia increases CD47 expression in CSCC cells. FCM analysis showing the expression of CD47 in normoxic/hypoxic SiHa and C33a cells. *, P<0.05. MFI, mean fluorescence intensity. [file 12964_2023_1450_MOESM4_ESM.jpg]

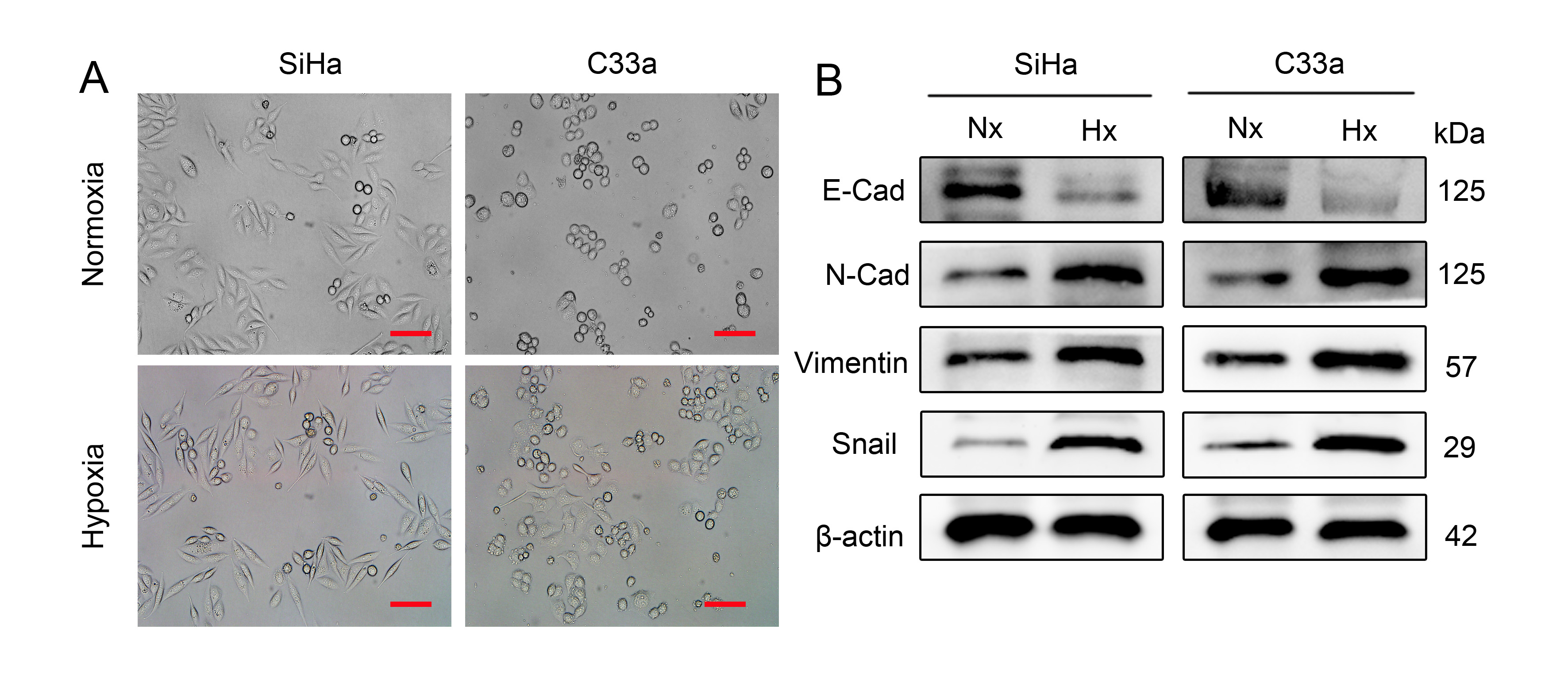

Supplement: Supplementary file 5 — Additional file 5: Supplemental Figure 2. Hypoxia promotes EMT in CSCC cells. [file 12964_2023_1450_MOESM5_ESM.jpg]

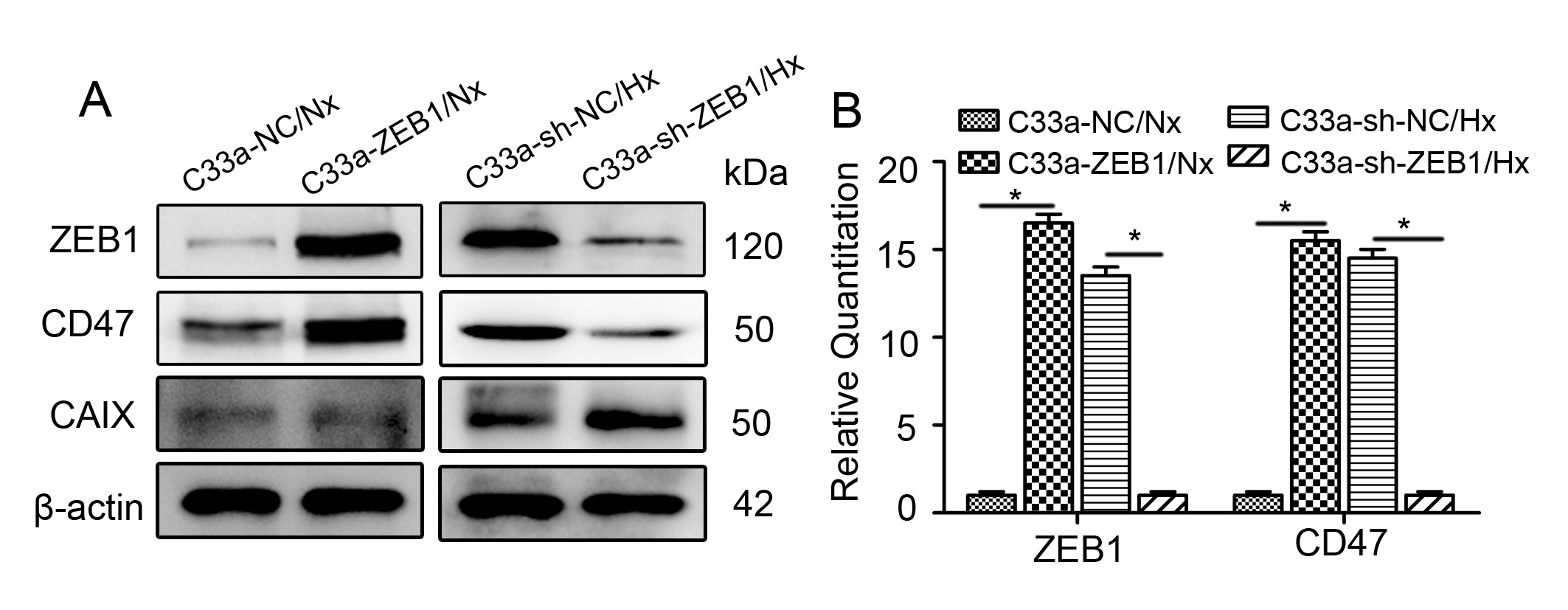

Supplement: Supplementary file 6 — Additional file 6: Supplemental Figure 3. Hypoxia-induced ZEB1 increases CD47 expression in C33a cells. [file 12964_2023_1450_MOESM6_ESM.jpg]

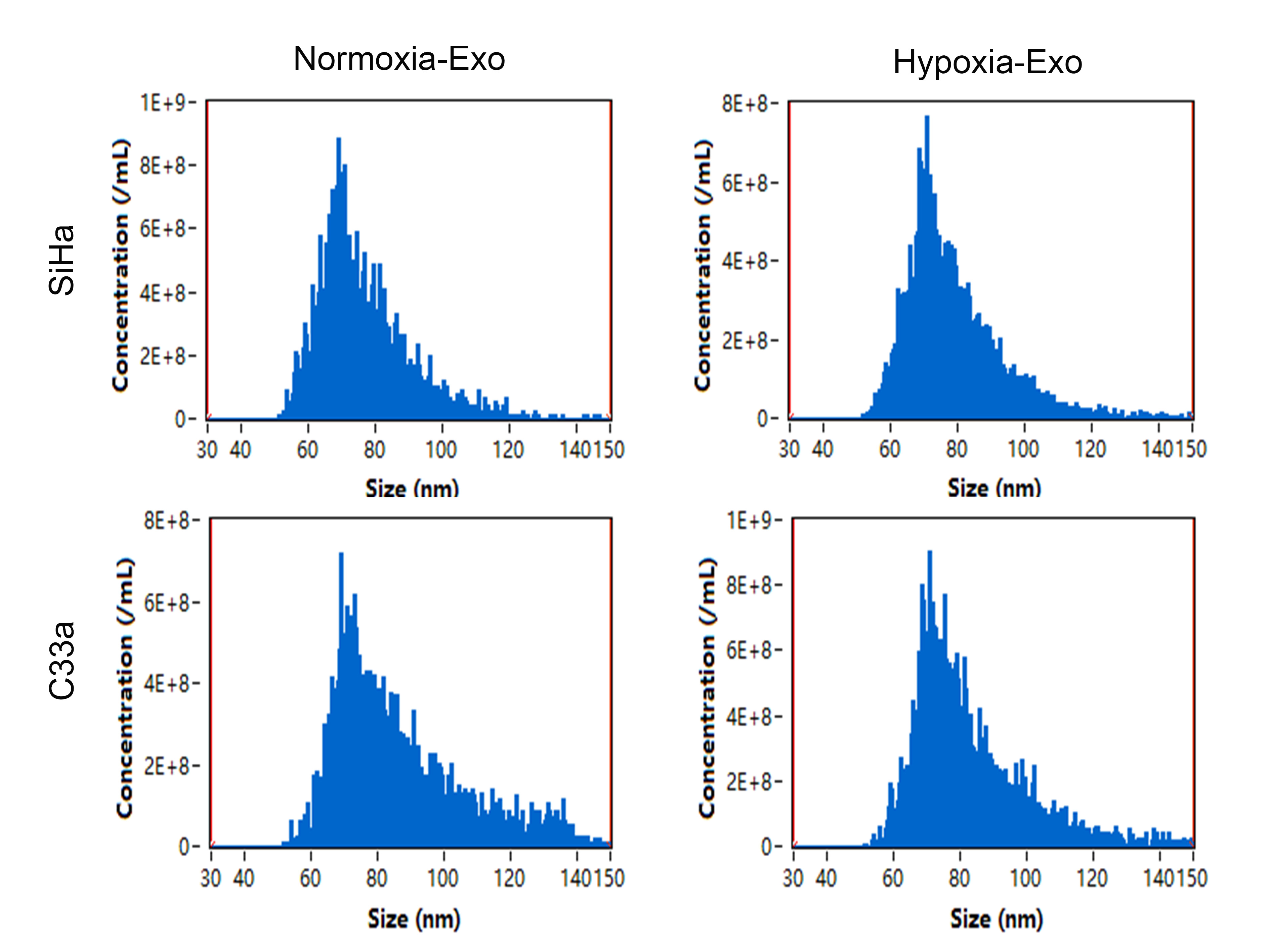

Supplement: Supplementary file 7 — Additional file 7: Supplemental Figure 4. Exosome size identification by nanoparticle tracking analysis (NTA). Exosomes secreted by normoxic and hypoxic CSCC cells (SiHa and C33a) were analysed by the NanoSight system. [file 12964_2023_1450_MOESM7_ESM.jpg]

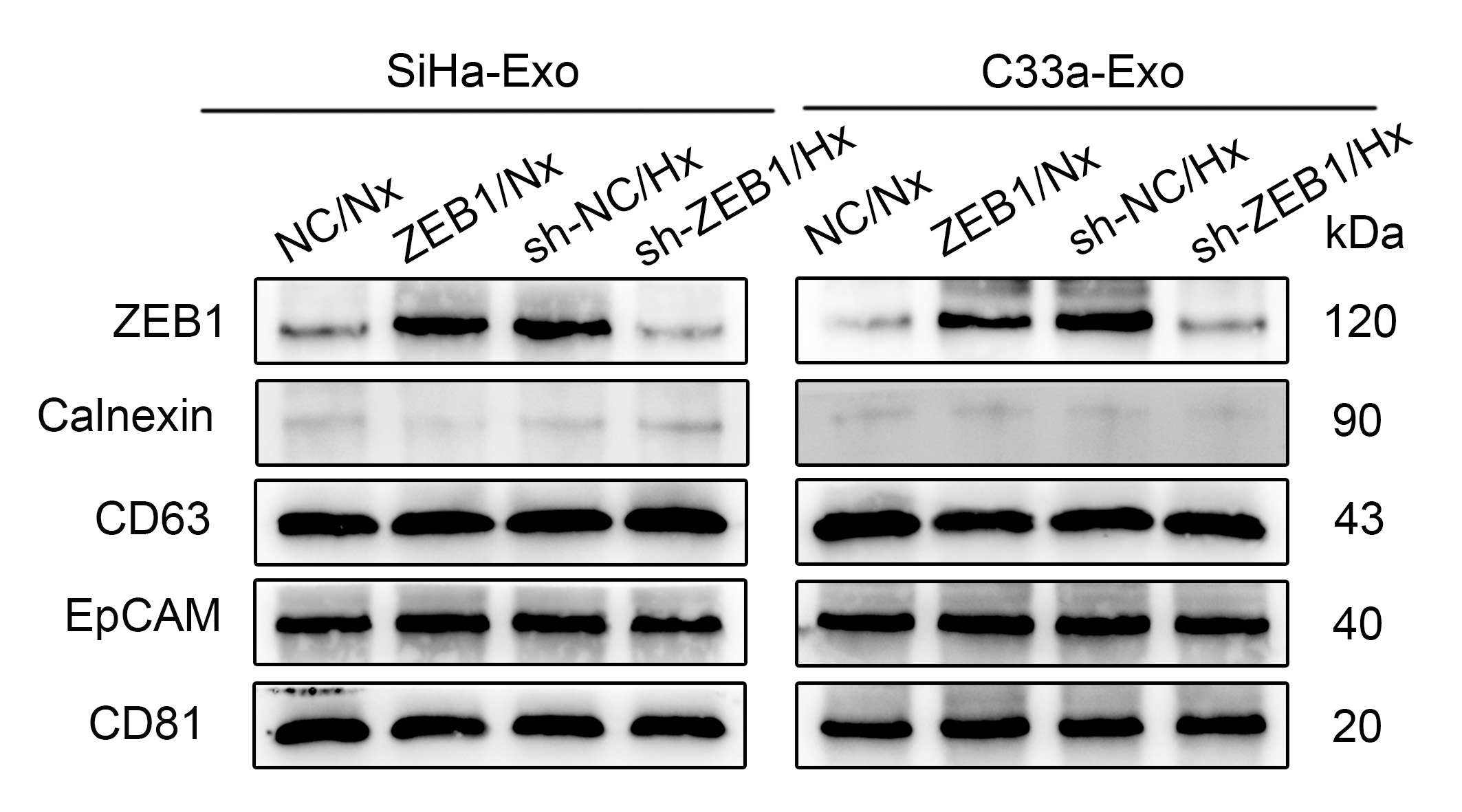

Supplement: Supplementary file 8 — Additional file 8: Supplemental Figure 5. ZEB1 expression levels in exosomes derived from CSCC cells. [file 12964_2023_1450_MOESM8_ESM.jpg]

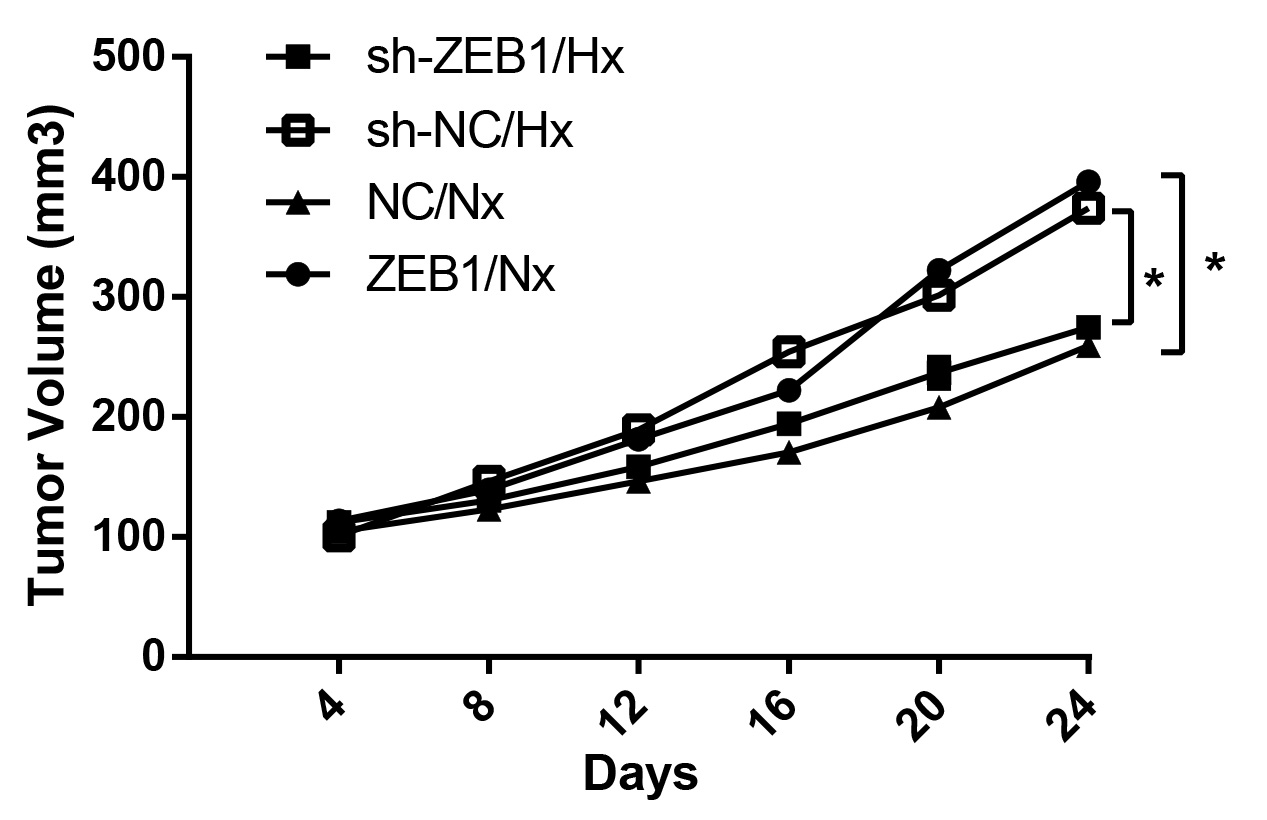

Supplement: Supplementary file 9 — Additional file 9: Supplemental Figure 6. Growth curve of CSCC xenograft model in vivo. *, P<0.05. Nx, normoxia; Hx, hypoxia; CSCC, cervical squamous cell carcinoma. [file 12964_2023_1450_MOESM9_ESM.jpg]
