# Supplementary material for: Hypoxia-induced ZEB1 promotes cervical cancer immune evasion by strengthening the CD47-SIRPα axis
Source: Cell Commun Signal. 2024 Jan 5;22:15. doi: 10.1186/s12964-023-01450-4 (PMC10768116; doi:10.1186/s12964-023-01450-4)
Supplement: Supplementary file 10 — Additional file 10: [file 12964_2023_1450_MOESM10_ESM.docx]

Full uncropped Gels and Blots images

Figure2B







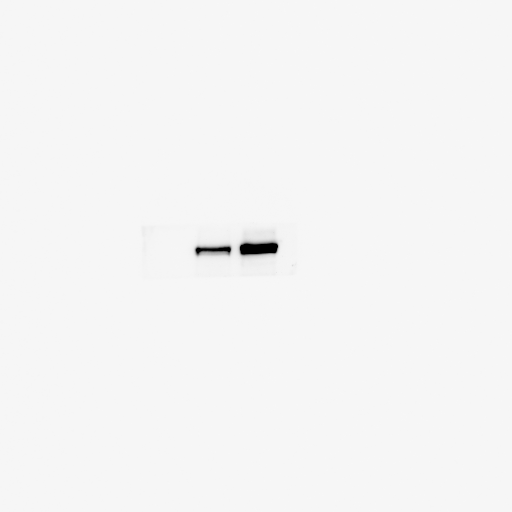














Figure3B



















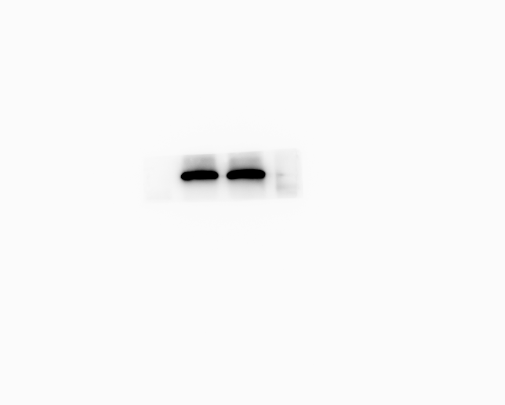







Figure3E












Figure4A






















Figure4B

















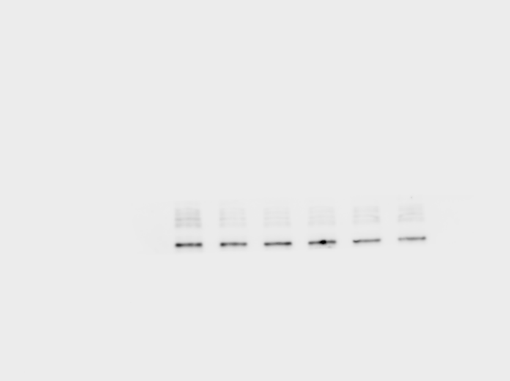





























Figure4C









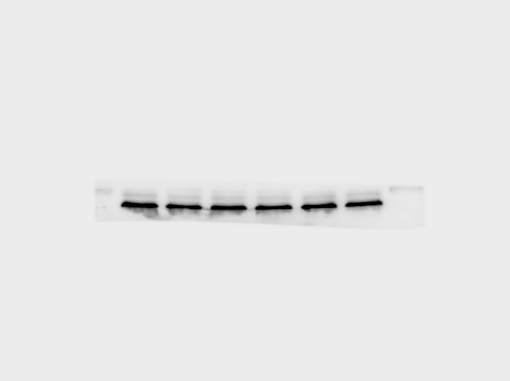

















Figure5C










Supplemental Figure2B

















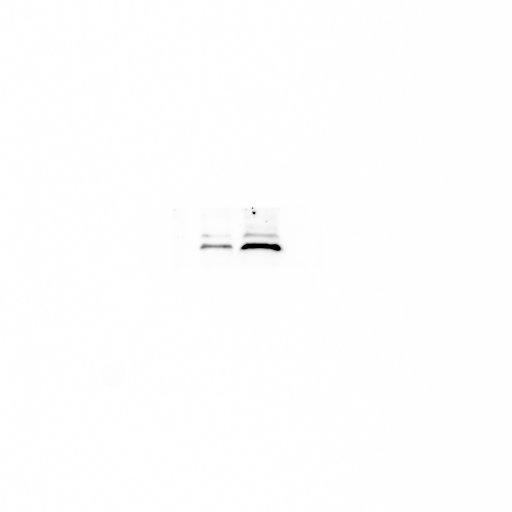

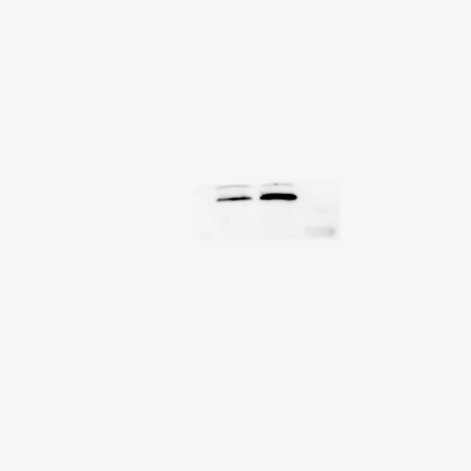


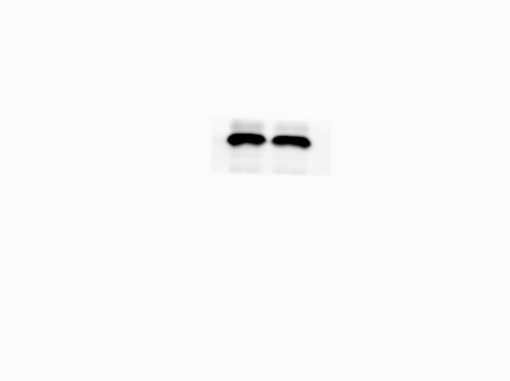




Supplemental Figure3A


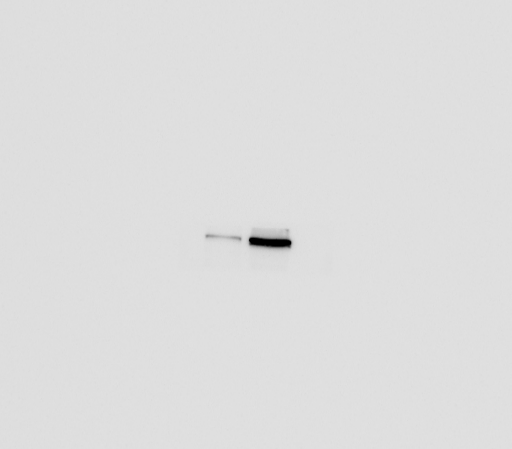

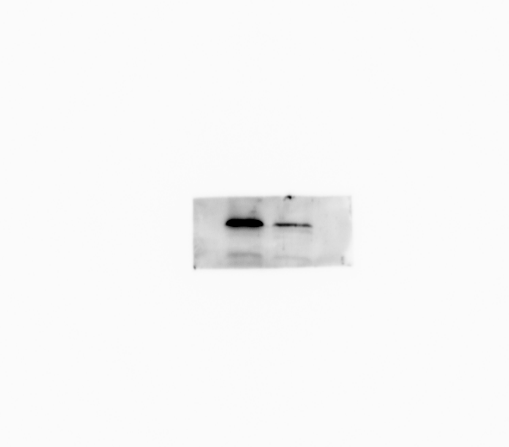


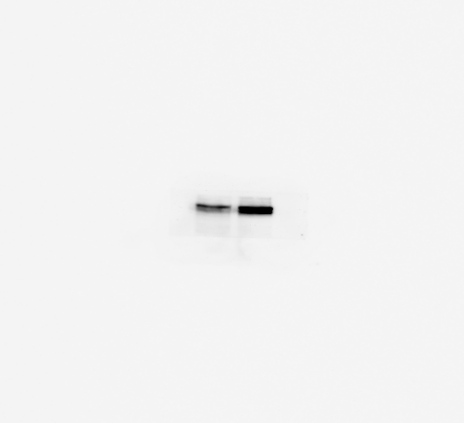














Supplemental Figure5
